# The Role and Diagnostic Efficacy of the METTL14/GADD45B m^6^A Methylation/BDNF Regulatory Axis in Acute Ischemic Stroke

**DOI:** 10.1007/s10571-026-01710-0

**Published:** 2026-04-16

**Authors:** Jin-ying Lai, Jun-hua Lu, Meng-yue Li, Bei-bei Li, Bao-yun Jin, Jiang-jie Hao, Ying Zhao, Ying Lin, Li-qiu Ma, Ren Liu, Shu-fan Zhang, Hong-jun Guan

**Affiliations:** 1 Department of Public Health, Mudanjiang Medical University, Mudanjiang, China; 2 Department of Nursing, Mudanjiang Medical University, Mudanjiang, China; 3 Hongqi Hospital Affiliated to Mudanjiang Medical University, Mudanjiang, China

**Keywords:** Acute ischemic stroke, N^6^-methyladenosine (m^6^A) methylation, Diagnosis, METTL14, GADD45B, BDNF

## Abstract

Despite advances in acute ischemic stroke (AIS) research, identifying reliable biomarkers and regulatory mechanisms remains challenging. We first identified AIS-related genes via extensive literature review, retrieved dataset GSE16561 from the Gene Expression Omnibus (GEO, https://ncbi.nlm.nih.gov/geo/), and performed differential/enrichment analyses. Bioinformatics verified N6-methyladenosine (m^6^A) sites in target genes, focusing on the methyltransferase-like protein 14 (METTL14)/growth arrest and DNA damage-inducible β (GADD45B) m^6^A methylation/brain-derived neurotrophic factor (BDNF) axis. Peripheral blood samples, biochemical indicators, and demographic data were collected from Hongqi Hospital Affiliated to Mudanjiang Medical University. Human umbilical vein endothelial cells (HUVECs) underwent transfection and oxygen-glucose deprivation (OGD). METTL14, GADD45B, and BDNF were detected by reverse transcription-quantitative polymerase chain reaction (RT-qPCR); BDNF protein by enzyme-linked immunosorbent assay (ELISA); global RNA m^6^A by Dot Blot; and GADD45B m^6^A by methylated RNA immunoprecipitation-quantitative polymerase chain reaction (MeRIP-RT-qPCR). Differential, diagnostic efficacy, logistic regression analyses, and nomogram validation were conducted. AIS patients showed increased METTL14, decreased GADD45B/BDNF, and increased levels of global m^6^A RNA and GADD45B m^6^A mRNA (*P* < 0.05). Receiver operating characteristic (ROC) analysis confirmed the three genes’ good diagnostic efficacy. The nomogram integrating these genes, globulin (GLOB), diabetes, high-density lipoprotein cholesterol (HDL-C), and hypertension performed excellently. This study highlights METTL14, GADD45B, and BDNF as key AIS biomarkers; METTL14 may indirectly regulate BDNF via GADD45B m^6^A methylation, providing potential therapeutic targets and novel mechanistic insights.

**Graphical Abstract:**

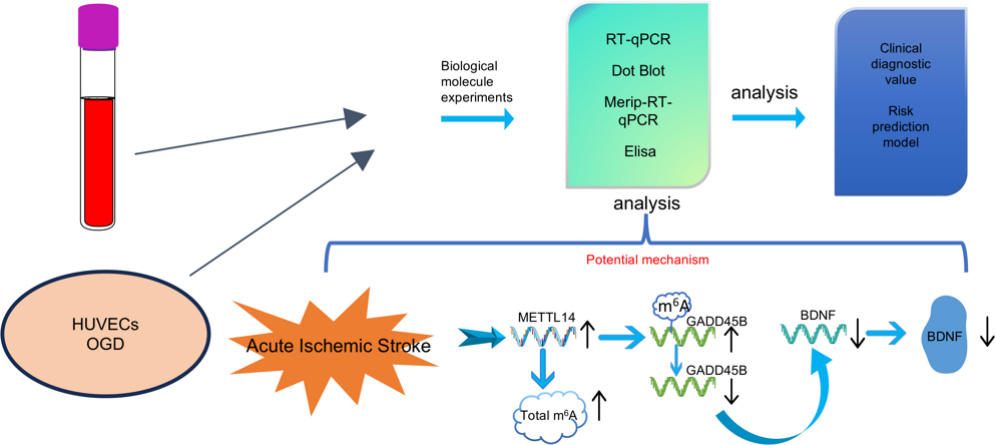

**Supplementary Information:**

The online version contains supplementary material available at 10.1007/s10571-026-01710-0.

## Introduction

Globally and in China, the incidence of stroke has been steadily increasing, making it one of the leading diseases that threaten human health. Among all stroke subtypes, AIS is characterized by high incidence, high recurrence rate, high disability rate, and high mortality, imposing a substantial burden on individuals, families, and society at large (Shi et al. 2024; Zhang et al. 2019, 2025b; Feng et al. 2025; Gao et al. 2023). While traditional risk factors, such as hypertension, smoking, and diabetes, partially explain the pathogenesis of AIS and its poor prognosis, a large number of underlying mechanisms remain unelucidated (Landete et al. 2023; Kumar et al. 2022; Xie et al. 2024). Notably, there are no well-validated biological diagnostic markers for AIS (Fu et al. 2021). Current diagnostic approaches primarily rely on computed tomography (CT) and magnetic resonance imaging (MRI); however, these modalities are associated with high costs and limited accessibility, as they are typically utilized in high-risk populations. Consequently, the general public often lacks awareness of early prevention strategies for AIS (Zheng et al. 2022; Yang et al. 2024).In terms of treatment, options include thrombolytic therapy, surgical intervention, antiplatelet drugs, and mechanical thrombectomy; however the overall prognosis of patients with AIS remains unsatisfactory (Li et al. 2023b; Chen et al. 2024). Collectively, clarifying the novel molecular mechanisms driving stroke pathogenesis and identifying key targets for prevention and treatment are of great significance for early prevention and effective management of stroke.

In recent years, epigenetic modifications have attracted extensive attention, particularly N6-methyladenosine (m^6^A) methylation of RNA, have become the most abundant post-transcriptional modification in mammals (Qiu et al. 2022; Liu et al. 2013). Similar to 5-methylcytosine (m^5^C), m^6^A methylation is a dynamic biochemical process involving three core components: “writers” (methyltransferases), “erasers” (demethylases), and “readers” (m^6^A-binding proteins). This modification is widely present in messenger RNAs (mRNAs) and non-coding RNAs (ncRNAs) of eukaryotes (Gao et al. 2024; Lu et al. 2024).

Studies have demonstrated that m^6^A modification is highly enriched in the mammalian brain and is closely associated with neurological disorders such as Alzheimer’s disease (AD) and Parkinson’s disease (PD) (Zhang et al. 2022; Lv et al. 2023). Importantly, accumulating evidence indicates that AIS can alter the cerebral m^6^A epitranscriptome, which may have functional implications for pathophysiological processes following AIS (Zhang et al. 2025c; Liu et al. 2023).

The major m^6^A methyltransferases include METTL14, Methyltransferase Like 3 (METTL3), Wilms Tumor 1-Associated Protein (WTAP), VIR-like m⁶A methyltransferase-associated protein (VIRMA), and Methyltransferase Like 16 (METTL16), while demethylases such as Fat Mass and Obesity-Associated Protein (FTO) and ALKBH5 can enzymatically reverse m^6^A modification (Xu et al. 2022). Li et al. (2023a) reported that METTL14 promotes M1 polarization and the NOD-Like Receptor Pyrin Domain Containing 3 (NLRP3) inflammasome/pyroptosis axis through the KAT3B-Stimulator of Interferon Genes (STING) signaling pathway following oxygen-glucose deprivation/reperfusion (OGD/R). Furthermore, depletion of METTL14 alleviated cerebral injury in middle cerebral artery occlusion (MCAO) rats by inhibiting M1-like microglia/macrophage polarization and the NLRP3 inflammasome/pyroptosis axis. Similarly, Liang et al. (2025) found that knockdown of METTL14 suppressed inflammation and pyroptosis, enhanced proliferation in OGD/R-treated microglia and primary microglia, and ameliorated cerebral ischemic injury in a rat MCAO model. Collectively, these findings indicate that METTL14 depletion can mitigate MCAO-induced cerebral injury and highlight a close association between METTL14 and m^6^A modification. However, studies investigating METTL14 and m^6^A modifications in human AIS tissues and cells have not yet been reported.

Growth Arrest and DNA Damage-Inducible Protein 45 Beta (GADD45B), also known as GADD45BETA or MYD118, belongs to the GADD45 family, which further includes Growth Arrest and DNA Damage-Inducible Alpha (GADD45A) and Growth Arrest and DNA Damage-Inducible Gamma (GADD45G). This family is associated with a variety of diseases, such as cancer and neurological disorders (Memarzia et al. 2023). GADD45B is a gene that plays critical roles in multiple biological processes, particularly DNA repair and demethylation, cell cycle regulation, senescence, and stress response (Zhang et al. 2021; Verzella et al. 2022). In the context of neuroprotection, the expression and function of GADD45B are tightly regulated and are essential for maintaining the stability and function of the nervous system (Zhang et al. 2025a). Notably, GADD45B may serve as a therapeutic target for AIS and harbors N6-methyladenosine (m^6^A) modification sites (Deng et al. 2021).

Brain-Derived Neurotrophic Factor (BDNF) is one of the most extensively studied neurotrophic factors and has drawn attention owing to its key roles in neuronal growth, differentiation, and survival (Huang et al. 2021). Changes in BDNF expression are closely associated with neuronal protection and repair (Gao et al. 2022; Wang et al. 2024). Liu et al. (2015) demonstrated that knockout of GADD45B in mice led to a decrease in BDNF gene expression, suggesting that GADD45B may regulate cell apoptosis by modulating BDNF and downstream apoptotic proteins in AIS. Thus, it can be inferred that the GADD45B promoter regulates DNA demethylation in the BDNF regulatory region.

In summary, AIS is a multifactorial disease and traditional diagnostic and therapeutic approaches have certain limitations, making the search for more effective targets for prevention/treatment and improved diagnostic methods an urgent priority. Based on previous literature, m⁶A modification is closely associated with IS, and METTL14, GADD45B, and BDNF may act as key biological factors in AIS. Therefore, the present study focused on the METTL14/GADD45B m^6^A methylation/BDNF regulatory axis to conduct experimental investigations.

## Methods and Materials

### Bioinformatics Analysis

First, datasets related to AIS were searched in the Gene Expression Omnibus (GEO) database (https://ncbi.nlm.nih.gov/geo/), and the GSE dataset consistent with the purpose of this study (GSE16561) was selected.

Using the limma package in R software (Version 4.5.1), differential expression analysis was performed to verify whether there was a significant difference in GADD45B expression between AIS and control samples. Volcano plots were generated using the ggplot2 package, and GSEA was conducted using the clusterProfiler package. Subsequently, the nucleotide sequence of GADD45B mRNA was downloaded from the National Center for Biotechnology Information (NCBI) database and submitted to the SRAMP server (http://www.cuilab.cn/sramp) to predict the N6-methyladenosine (m^6^A) modification sites on GADD45B mRNA (Elsabbagh et al. 2025).

Additionally, the RM2Target platform (http://rm2target.canceromics.org/citation) was used to predict the potential of METTL14 to regulate GADD45B mRNA (Xu et al. 2023). Similarly, the nucleotide sequence of GADD45B mRNA and the protein sequence of BDNF were downloaded from NCBI and uploaded to the RPISeq server (http://pridb.gdcb.iastate.edu/RPISeq/) to predict the possibility of an interaction between GADD45B mRNA and BDNF protein (Quan et al. 2025).

### Sample and Clinical Information Collection

From September 2024 to June 2025, patients with AIS were recruited as the case group, and healthy individuals who underwent physical examinations during the same period were enrolled as the control group at Hongqi Hospital Affiliated to Mudanjiang Medical University, Heilongjiang Province. All participants provided informed consent before participating in the study. This clinical study was approved by the Institutional Review Board (IRB) of Hongqi Hospital Affiliated to Mudanjiang Medical University (approval number: 2025-MXSZR07). The onset time of AIS is within 6 h to 7 days (Park et al. 2020), prior to the collection of peripheral blood samples, biochemical indices, and demographic data, all participants (or their legally authorized representatives if applicable) were fully informed of the study purpose, procedures, potential risks, and benefits. Written informed consent was obtained from each participant and the study was conducted in compliance with the Declaration of Helsinki.

A 1:1 matched case-control study design was adopted, where volunteers in the Control group were selected to match those in the case group by the same sex and an age difference of no more than three years.

Inclusion criteria for the case group met the diagnostic criteria of the Chinese Guidelines for the Diagnosis and Treatment of Acute Ischemic Stroke, confirmed as AIS by CT/MRI, with etiological classification conforming to the TOAST criteria: aged 18–90 years; written informed consent provided by patients or their legal representatives, who agreed to participate in medical history collection and sample provision; and complete data available, including demographic information, medical history, and biochemical indices.

Exclusion criteria for the case group were as follows: suspected or confirmed hemorrhagic stroke, subarachnoid hemorrhage, or intracranial vascular malformation/aneurysm indicated by imaging; complications with malignant tumor, severe hepatic/renal failure, coagulation disorders, or history of intracranial surgery/severe head trauma within 3 months; severe lack of clinical data, or inability to cooperate with the study due to cognitive/psychiatric disorders, pregnant or lactating women, or participation in other investigational drug/device studies within 30 days.

Inclusion criteria for the Control group were as follows: no history of AIS; cerebrovascular diseases and other organic neurological lesions excluded by physical examination and cranial imaging; 1:1 matched with cases (age ± 3 years, same sex), recruited from the same study population (concurrent non-cerebrovascular inpatients or community-dwelling healthy individuals); provided informed consent and agreed to blood sample and clinical data provision; and no severe comorbidities affecting study indices (uncontrolled hypertension, diabetes, etc., were allowed to ensure baseline balance).

Exclusion criteria for the Control group were as follows: history of transient ischemic attack (TIA) or cerebral infarction, or cerebrovascular stenosis > 50% indicated by imaging; complicated with coagulation abnormalities, autoimmune diseases, or recent use of drugs affecting inflammatory/metabolic indices (e.g., long-term glucocorticoids); and met the criteria for severe comorbidities, special conditions, or poor cooperation as excluded in the case group.

Baseline data and relevant blood biochemical indicators were collected from all the participants. For each participant, 5 mL of fasting venous blood was aseptically collected in the morning in EDTA anticoagulant tubes. From this blood sample, 1 mL of whole blood was centrifuged at 2500 rpm and 4 °C for 20 min to obtain plasma. The remaining whole blood was mixed with the TRIzol reagent at a volume ratio of 1:5. All processed samples were aliquoted and immediately stored in a −80 °C ultra-low-temperature refrigerator for subsequent experiments. A pilot RT-qPCR experiment was conducted using 25 sample pairs, focusing on the relative expression levels of the three target genes. The sample size was calculated using the formula ${\mathrm{n}}~ = \frac{{2 \times ({\mathrm{Z}}_{{\alpha /2}} + {\mathrm{Z}}_{\beta } )^{2} \times \sigma ^{2} }}{{(\mu _{1} - \mu _{0} )^{2} }}$, where α = 0.05 and power = 95%. The calculated sample sizes were 55, 162, and 59. The maximum value (162) was adopted, indicating that a minimum of 162 sample pairs was required.

### Cell Experiments

#### HUVEC Culture

Human umbilical vein endothelial cells (HUVECs) were purchased from Wuhan Punosai Biotechnology Co., Ltd. The entire cell culture process was conducted in a laminar flow hood sterilized with 75% alcohol and ultraviolet light. Prior to experiments, culture media, digestive enzymes, PBS, and other reagents were balanced to room temperature. Cell suspension counting was performed using a cell counter, and cell density was adjusted according to experimental requirements. After experiments, the laminar flow hood was cleaned to ensure a sterile experimental environment. For this experiment, cells were cultured in T_25_ culture flasks (LABSELECT, Cat# 13112 A, China). The primary tools for modeling and transfection were 6-well plates (LABSELECT, Cat# 11110, China), while the CCK8 assay utilized 96-well plates (LABSELECT, Cat# 11510, China). Dulbecco’s Modified Eagle Medium (DMEM, Biosharp, Cat# BL305A, China), Penicillin-Streptomycin Solution (Biosharp, Cat# BL505A, China), Fetal Bovine Serum (FBS, Biosharp, Cat# BL201A, China), Dimethyl sulfoxide (DMSO, Biosharp, Cat# BS087-100 mL, China). The complete medium was composed of 89% DMEM, 10% FBS, and 1% Penicillin-Streptomycin Solution, while the cryopreservation solution consisted of 50% complete medium, 40% FBS, and 10% DMSO.

Cell Resuscitation: Remove the frozen cells from the−80 °C refrigerator and rapidly thaw them in a 37 °C water bath for approximately 2 min until fully dissolved. Transfer the thawed cell suspension to a centrifuge tube and resuspend with 1–2 mL of complete medium. Centrifuge at 1000 rpm for 3 min at room temperature, discard the supernatant, and retain the cell pellet. Subsequently, resuspend the cells in 1–2 mL of complete medium. Aspirate the cell suspension and evenly distribute it into pre-prepared culture dishes. Gently shake the dishes to ensure uniform cell distribution. After labeling, incubate in a 37 °C, 5% CO_2_ incubator.

Cell Passaging: Regularly monitor cell growth status. When the cells exhibit optimal morphology and fully cover the culture dish bottom, perform passaging. Aspirate the medium from the dish and gently rinse the cells three times with 2–3 mL of PBS buffer. Add 1mL of trypsin digestion solution and incubate at 37 °C in a 5% CO_2_ incubator for 3 min. Based on microscopic observation of cellular morphology changes, add 1 mL of complete medium to terminate digestion. Gently vortex the cell suspension to homogenize, transfer to a centrifuge tube, and centrifuge at 1000 rpm for 3 min. Discard the supernatant, add 2 mL of fresh complete medium to the cell pellet, and mix thoroughly. Uniformly inoculate the cell suspension into a new culture dish. Supplement with an appropriate amount of complete medium, label, and continue incubation at 37 °C in a 5% CO_2_ incubator.

Cell Counting: Prepare a cell suspension using the automated cell counter method (Countess), and mix it uniformly with trypan blue staining solution at a 1:1 ratio. Add a small amount of the mixture to the dedicated detection chip of the counter. Load the sample into the counter, select the corresponding detection mode, and the instrument will automatically complete the counting and display data such as cell concentration and viability percentage.

Cell Cryopreservation: Remove the old medium from the culture dish and gently rinse the cells three times with 1–2 mL of PBS buffer. Then, add 1mL of trypsin digestion solution and incubate at 37 °C in a 5% CO_2_ incubator for 3 min. Based on microscopic observation of cellular morphology changes, add 1 mL of complete medium to terminate digestion. Transfer the cell suspension to a centrifuge tube and centrifuge at 1000 rpm for 3 min. Discard the supernatant and retain the cell pellet. Add an appropriate amount of 2 mL cell cryopreservation solution to the pellet and gently vortex to mix. Transfer the uniformly mixed cell suspension into cryovials, label them, and place them in a programmed cooling box before transferring to a−80 °C freezer for storage.

### Construction of a HUVEC Model of OGD

Cell seeding: On the day before the experiment, cells were grouped according to experimental requirements and inoculated into 6/96-well culture plates at the predetermined cell density. The plates were then incubated in a 37 °C, 5% CO_2_ incubator for 24 h to ensure full cell adhesion.

OGD treatment: Discard the old medium, wash the cells with PBS buffer three times, add an appropriate amount of sugar-free DMEM medium (Biosharp, Cat# BL304A, China) to the culture plate, and transfer the cells to a incubator at 37 °C, 1% O_2_, 5% CO_2_, and 94% N_2_ for 6 h of hypoxia treatment (Li et al. 2021). The Control group cells were not subjected to OGD treatment and were cultured in a 37 °C, 5% CO_2_ incubator using complete medium to maintain normal cell growth.

### CCK-8 Assay for Viability of OGD-Treated HUVECs

Cell seeding: Inoculate cells into a 96-well plate and incubate for 24 h until adhesion and stabilization.

OGD treatment: The OGD group was switched to a sugar-free medium and incubated in a hypoxic incubator (1% O₂) (Thermo Fisher Scientific, Massachusetts, USA) for 6 h. The Normal group was maintained in a normal medium under normoxic conditions, with the same treatment duration as the OGD group.

Normal group: Maintain normal medium and oxygen conditions, with treatment duration consistent with the OGD group.

CCK-8 assay: Replace with a new complete medium and add 10% volume of CCK-8 reagent per well. Incubate at 37 °C in a dark place for 2 h. Measure the absorbance at 450 nm (OD value) using an enzyme-labeled instrument.

The success criterion of the OGD model was defined as a significant reduction in cell viability, specifically a marked decrease in the survival rate of cells in the OGD group (expressed as the OD value relative to the Normal group).

### Transfection of HUVECs

Cell seeding: On the day prior to transfection, cells were inoculated into a 6-well plate. Using 2 mL of DMEM complete medium per well as the standard, 1.5 × 10^5^ cells were seeded and incubated at 37 °C in a 5%CO_2_ incubator for 24 h to achieve cell adhesion and optimal density.

Transfection reagent preparation: The transfection reagent was divided into two groups, A and B, for preparation. Group A: Take 200 µL of opti-MEM medium and add it to a sterile EP tube. Then, add 5 µL of Lipofectamine 2000 transfection reagent (EallBio, Cat# 03.17001DA, China), gently mix, and incubate in the dark for 5 min. Group B: Take another 200 µL of opti-MEM medium (EallBio, Cat# 03.18001DA, China) and add it to a new sterile EP tube. Add 5 µL of SiRNA master solution, mix, and incubate in the dark for 5 min.

Preparation of transfection complex: The liquid from tube A was slowly transferred to tube B, gently mixed, and then left undisturbed in the dark for 20 min to allow full formation of the transfection complex.

Transfection procedure: Discard the old medium in the 6-well plate and add 1600 µL of opti-MEM medium per well to minimize the effect of serum on transfection efficiency. Uniformly add the prepared transfection complex to each well of the 6-well plate, gently shaking the plate to ensure even distribution of the solution. Incubate the cells in a 37 °C, 5% CO_2_ incubator for 4–6 h. After incubation, discard the transfection complex and replace it with fresh DMEM complete medium, then continue the culture for 24 h.

According to experimental requirements, cells were collected at 24–48 h post-transfection for subsequent experiments. Table 1 is a list of SiRNA and NC-siRNA sequences.

**Table 1:** Transfection sequence

Oligo name	Sequence (5’ to 3’)
Si-METTL14	Sense GGAUGAAGGAGAGACAGAUUU
	Anti-sense AUCUGUCUCUCCUUCAUCCUU
Si-GADD45B	Sense ACAUCUCUCUUCAGGAACGCUUU
	Anti-sense AGCGUUCCUGAAGAGAGAUGUUU
NC-siRNA	Sense UUCUCCGAACGUGUCACGU
	Anti-sense ACGUGACACGUUCGGAGAA

### RT-qPCR

Total RNA was extracted from whole blood using TRIzol reagent. The quality of the extracted RNA was verified by measuring the absorbance ratios; the A260/A280 ratio was required to be within the range of 1.9–2.1, and the A260/A230 ratio within 1.9–2.2, to ensure no contamination by genomic DNA, proteins, inorganic salts, or organic compounds. Subsequently, complementary DNA (cDNA) was synthesized via reverse transcription using HyperScript™ III RT SuperMix for qPCR with a gDNA Remover kit (EnzyArtisan, Cat# R202, China) (Negi et al. 2025). Glyceraldehyde-3-phosphate dehydrogenase (GAPDH) was used as the internal reference gene and 2×S6 Universal SYBR qPCR Mix (EnzyArtisan, Cat#Q204, China) was used for quantitative PCR (qPCR) amplification. Primers for METTL14, BDNF, and GAPDH were designed using Primer-BLAST, whereas primers for GADD45B were obtained by reviewing a large number of relevant studies. Primer-BLAST was used not only for primer design but also to verify primer specificity and amplicon size. All primers were synthesized by Shanghai Biological Engineering Co. Ltd. The qPCR reaction system was 10µL, with the following reaction conditions: an initial pre-denaturation step at 95 °C for 30 s (one cycle), followed by 45 cycles of denaturation at 95 °C for 10 s, and annealing extension at 60 °C for 30 s. The 2^⁻ΔΔCT^ method was used to calculate the relative quantification of target gene expression.

### Dot Blot

Apply 2 µL of RNA (400 ng/µL) to each point. After quantification and denaturation (95 °C, 5 min), RNA samples were loaded onto a positively charged nylon transfer membrane (LABSELECT, Cat# TM-NY-S-45, China). The RNA was cross-linked to the membrane by ultraviolet (UV) irradiation for 90 min. The film was rinsed with TBST (Biosharp Cat# BL1683A, China), and one film was removed as an internal standard. It was incubated in methylene blue solution (Solubar Cat# G1305, China) on a shaker for 5 min, and a photograph was taken as a control. Subsequently, another membrane was washed with Tris-buffered saline solution containing Tween-20 (TBST; Biosharp, catalog number BL1683A, China) and blocked with 5% non-fat milk (Biosharp, catalog number BS102-100 g, China) at room temperature for 1 h. After blocking, the membrane was incubated overnight at 4 °C with a primary antibody against m^6^A (1:800; ABclonal, Cat# A17924, China, Harbin Songbei Huajiekai Chemical & Biotechnology Distribution Department, RRID: AB_2770239), followed by recognition with a secondary antibody (1:6000; ABclonal, Cat# AS014, China, Harbin Songbei Huajiekai Chemical & Biotechnology Distribution Department, RRID: AB_2769854). The specificity of the m^6^A rabbit polyclonal antibody (A17924, ABclonal) was validated using ABclonal via multidimensional experiments (see Supplementary File 3). Both primary and secondary antibodies were diluted in a 5% non-fat milk solution prepared with TBST (Li et al. 2023a, b). After thorough washing to remove unbound antibodies, the membrane was incubated with Enhanced Chemiluminescence (ECL, Biosharp, Cat# BL523A, China) reagent in the dark for approximately 1 min, followed by immediate visualization and imaging using a chemiluminescence imaging system. Images of the membranes were captured after this step and served as a loading control. Relative content analysis was performed using ImageJ v1.8.0. The resulting calculation method was as follows: m^6^A gray value/MB gray value (Du et al. 2022; Xu et al. 2023).

### MeRIP-RT-qPCR

For each sample, the total RNA input amount was 4 µg in the immunoprecipitation (IP) group and 200 ng in the Input group. Methylated RNA immunoprecipitation (MeRIP) assay was performed using the EpiQuik CUT&RUN m^6^A MeRIP Kit (Cat# P-9018-24, USA).

The MeRIP procedure can be summarized as follows: Total RNA was incubated with m⁶A antibody-conjugated magnetic beads and m^6^A antibody for 90 min to capture RNA containing m^6^A modifications. Subsequently, the captured RNA was fragmented using CEM (Calcium- and Magnesium-containing) buffer and washed; thereafter, the RNA was incubated with RNA Binding Beads for 5 min to bind and recover the enriched RNA. The m^6^A-modified RNA fragments were then released and dissolved using Elution Buffer, with all specific steps strictly performed according to the kit instructions. The eluted m^6^A-modified RNA fragments were immediately reverse-transcribed into complementary DNA (cDNA) at an equal volume and stored at − 80 °C for subsequent RT-qPCR analysis. A normalized Input group was used as the reference, and the 2^⁻ΔCT^ method was applied to calculate the relative percentage of m^6^A methylation of GADD45B and conduct statistical analysis.

### ELISA

Plasma extraction: Centrifuge the sample at 4 °C and 2500 rpm for 20 min within 30 min after collection, collect the supernatant, and store at −80 °C.

Preparation of HUVECs extract: Adherent HUVECs were gently washed twice with cold PBS, followed by 1 mL trypsin digestion for 3 min. After adding 2 mL complete medium to terminate digestion, samples were collected and counted. Cells were then collected by centrifugation at 2000 rpm for 5 min. The collected cells were washed three times with cold PBS. A suspension of 200 µL PBS per 1 × 10^6^ cells was prepared, and the cells were lysed by repeated freeze-thaw cycles at −20 °C to 37 °C for three times. The suspension was then centrifuged at 2000 rpm for 10 min, and the supernatant was collected for storage at − 80 °C for subsequent experiments.

Prior to the experiment, all plasma, cell extracts, and reagents were equilibrated at room temperature. Specifically, the experiment required setting up standard wells, sample wells, and blank wells, and measuring the optical density (OD) values of each well at a wavelength of 450 nm. All experimental steps were strictly followed according to the instructions of the ELISA kit (Changchun Shuo, product number ED-12065,96-well plate size, China).Steps to reduce subjective bias.

### Materials

Supplement consumables: Sterile and enzyme-free blood collection tubes (Kangshifei, Cat# 367863 K2EDTA, China), 2.0 mL Cryogenic vials (LABSELECT, Cat# CV-002–200-EX, China) Trizol (Biosharp, Cat# BS258A, China), Chloroform (Damao, AR grade, China), Isopropanol (≥ 99.5%, Merck KGaA, Darmstadt, Germany), Absolute ethanol (≥ 99.5%, Sigma-Aldrich, St. Louis, MO, USA), DEPC-treated water (Biosharp, Cat# BL510A, China), 1.5 mL Microcentrifuge Tube (LABSELECT, Cat# MCT-001–150, China), 10 mL Microcentrifuge Tube (BKMAMLAB, Cat# 110403052, China), 0.2 mL flat-cap 8-strip PCR tubes (Cat# PCR-0208-C) and matching 8-strip sealing caps (Cat# PCR-2CP-RT-C) were purchased from Axygen Scientific (Union City, CA, USA), 0.2 mL flat-cap thin-wall PCR tubes (Axygen Scientific, Cat# PCR-02-C, Union City, CA, USA).

Related equipment: refrigerator (4 °C; Haier, Qingdao, China), freezer (− 20 °C; Haier, Qingdao, China), ultra-low temperature freezer (− 80 °C; Haier, Qingdao, China), refrigerated centrifuge (4 °C; TOMY, Cat# MX-307, Japan), ultramicro nucleic acid and protein detector (Thermo Fisher Cat# Nanodrop 2000, USA), Conventional PCR thermocycler (Bio-Rad, Cat# T100, USA), Applied Biosystems 7500 Fast Real-Time PCR System (Thermo Fisher, USA), chemiluminescence imaging system (Thermo Fisher, Cat# iBright FL1500, USA).

### Statistical and Data Reporting Guidelines

All data in this study were analyzed using SPSS software (version 27.0, IBM SPSS Statistics), with statistical significance set at *P* < 0.05. During statistical analysis, researchers employed a blinded design to avoid subjective bias. First, normality and homogeneity of variance tests were performed for all continuous data to determine the appropriate statistical analysis method, and continuous variables were presented as mean ± standard deviation if normally distributed or median with interquartile range [*M* (*P*_25_, *P*_75_)] if non-normally distributed. For paired samples, the paired t-test was used when the difference between the two groups conformed to a normal distribution, while the Wilcoxon signed-rank test was applied if the difference was non-normally distributed. Categorical variables were analyzed for differences using McNemar’s test, and all graphs were generated using GraphPad Prism (Version 10.1.2). Correlation analysis was conducted between METTL14, GADD45B, and BDNF. Pearson correlation analysis was used if the two groups of data were normal and the variance was equal, and Spearman correlation analysis was used if the two groups of data were not normal or the variance was not equal. Receiver operating characteristic (ROC) analysis was performed to evaluate the diagnostic efficacy of the three target genes. Factors without significant differences were excluded, and the remaining factors that potentially influenced AIS were analyzed using matched logistic regression. Variables with statistical significance in the univariate regression model were included in the multivariate logistic regression analysis to identify the key factors influencing AIS. A nomogram was constructed based on these key factors and its predictive efficacy was evaluated. The normality test and homogeneity test of variance were performed on the results of the cell experiment, and the appropriate method was selected for the analysis of differences.

## Results

### Clinical Data of AIS Group and Control Group

A total of 177 eligible matched sample pairs were collected from Hongqi Hospital, which is slightly more than the calculated 162. The age distributions of both the case and Control groups did not follow a normal distribution, as indicated by the Kolmogorov-Smirnov Test (case group: *D*_(177)_ = 0.113, *P*< 0.001; Control group: *D*_(177)_ = 0.082, *P* < 0.001; *P *> 0.05 indicates normality). The age of the case group was 65.00(60.00, 70.00) [*M* (*P*_25_, *P*_75_)], and that of the Control group was 66.00 (59.00, 69.00), with no statistically significant difference between the two groups (*Z*_(177)_ = − 1.849, *P* = 0.065). Among the sample pairs, 111 were male and 66 were female. The National Institutes of Health Stroke Scale (NIHSS) score of patients was 3.00(2.00,5.00), and the Kolmogorov-Smirnov Test result was *D*_(177)_ = 0.220, *P *< 0.001. Since they were paired samples, the Kolmogorov-Smirnov test was performed on the differences in baseline data and biochemical indicators between the IS group and Control group, with the following results: TP (*D*_(177)_ = 0.051, *P* = 0.200), ALB (*D*_(177)_ = 0.075, *P*= 0.016), GLOB (*D*_(177)_ = 0.035, *P* = 0.200), TC (*D*_(177)_ = 0.171, *P* < 0.001), HDL-C (*D*_(177)_ = 0.082, *P* = 0.005), LDL-C (*D*_(177)_ = 0.035, *P*= 0.200), and GLU (*D*_(177)_ = 0.062, *P* = 0.092). The paired-samples *t*-test was used for normally distributed data and the Wilcoxon signed-rank test was used for non-normally distributed data. As shown in Table 2, statistically significant differences ( *P*< 0.05) were observed in hypertension (χ^2^_(176)_ = 54.747, *P* < 0.001), diabetes (χ^2^_(176)_ = 39.585, *P*< 0.001), dyslipidemia (*χ*^2^_(176)_ = 3.905, *P*= 0.048), and biochemical markers, including TP (*t*_(176)_ = −7.343, *P* < 0.001), ALB (*Z*_(176)_ = −7.263, *P* < 0.001), GLOB (*t*_(176)_ = 5.294, *P* < 0.001), TC(*Z*_(176)_ = −2.386, *P* = 0.017), HDL-C(*Z*_(176)_ = −4.617, *P* < 0.001), LDL-C(*t*_(176)_ = −2.986, *P* = 0.003), and GLU(t_(176)_ = 6.121, *P* < 0.001). No statistically significant differences were observed between the two groups in triglyceride (TG) levels (*Z*_(176)_ = −1.153, *p* = 0.249), drinking status (*χ*^2^_(176)_ = 3.724, *P* = 0.054), or smoking status (*χ*^2^_(176)_ = 1.891, *P* = 0.169), and all other variables showed statistically significant differences.

**Table 2:** Baseline data and biochemical indicators of the AIS group and the control group

Variable	AIS (*n* = 177)	Control (*n* = 177)	*d*	*t*/*Z*/χ^2^	*P *
Age	65 (60,70)	66 (59,69)	–	−1.85	0.065
TP (g/L)	69.70 (65.50,73.60)	74.20 ± 4.43	−4.43 ± 8.03	−7.343	< 0.001***
ALB (g/L)	42.13 (38.79,44.86)	44.25 ± 2.90	−2.51 (4.86,−0.50)	−7.263	< 0.001***
GLOB (g/L)	31.79 ± 4.10	29.78 ± 3.93	2.01 ± 5.06	5.294	< 0.001***
GLU (mmol/L)	6.27 (5.30,8.09)	4.84 (4.47,5.33)	1.16 ± 2.52	6.121	< 0.001***
TG (mmol/L)	1.29 (0.93,1.90)	1.43 (1.06,2.01)	−0.01 (−0.08,0.60)	−1.153	0.249
TC (mmol/L)	4.51 (3.77,5.24)	4.89 ± 1.10	−0.24 (−1.33,0.77)	−2.386	0.017*
HDL-C (mmol/L)	1.05 (0.89,1.29)	1.21 (1.05,1.44)	−0.16 (−0.43,0.15)	−4.617	< 0.001***
LDL-C (mmol/L)	2.36 (1.86,2.94)	2.65 ± 0.83	−0.24 ± 1.10	−2.986	0.003**
Diabetes, n (%)	113 (63.84%)	51 (28.81%)	–	39.585	< 0.001***
Hypertension, n (%)	79 (44.63%)	11 (6.21%)	–	54.744	< 0.001***
Smoking, n (%)	55 (29.94%)	43 (24.29%)	–	1.891	0.169
Drinking, n (%)	70 (39.54%)	51 (28.81%)	–	3.724	0.054
Dyslipidemia, n (%)	136 (63.84%)	118 (66.67%)	–	3.905	0.048*
NHISS	3.00 (2.00,5.00)	–		–	–

*AIS* acute ischemic stroke; *d* the difference between the AIS group and the Control group; *IQR* interquartile range; *TP* total protein; *ALB* albumin; *GLOB* globulin; *GLU* glucose; *TG* triglycerides; *TC* total cholesterol; *HDL-C* high-density lipoprotein cholesterol; *LDL-C* low-density lipoprotein cholesterol; and *NIHSS* national institutes of health stroke scale, a *P*-value < 0.05 was considered statistically significant (*P < 0.05, **P < 0.01, ***P < 0.001), and "ns" was regarded as no statistical difference.

### The Expression Levels of METTL14/GADD45B/BDNF Exhibit Significant Differences Between the Control Group and the AIS Group

**Fig. 1:**
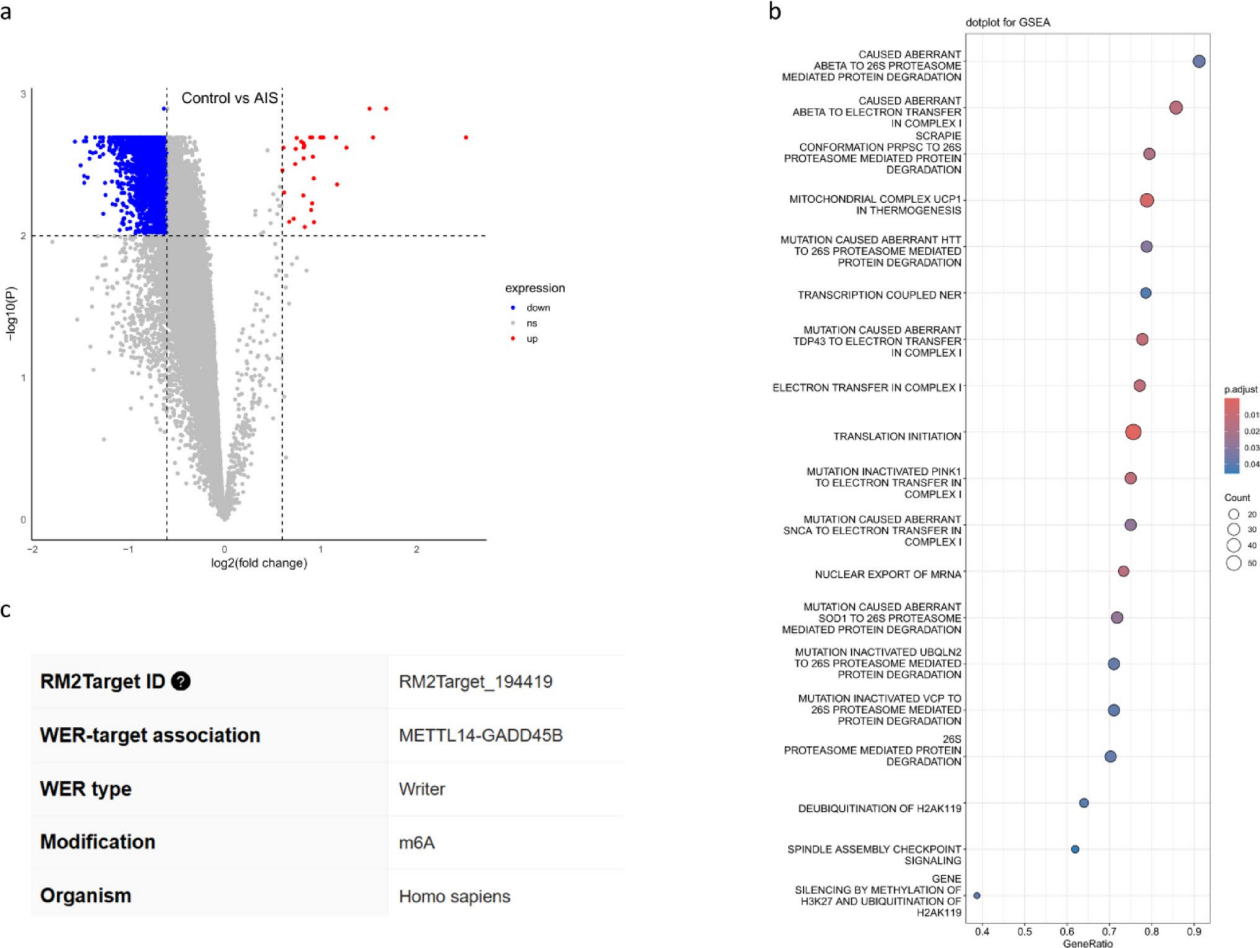
Bioinformatics analysis related to GADD45B.** a** Volcano plot of differential analysis. **b** GSEA functional enrichment analysis of GADD45B. **c** Prediction results from RM2Target

AIS-related datasets were searched in the Gene Expression Omnibus (GEO) database, and the GSE16561 microarray dataset was ultimately selected for analysis because it comprises human peripheral blood samples. To maintain consistency with our experimental design (matching by gender and age difference ≤ 3 years), seven sample pairs were selected and raw data were downloaded for analysis. Differential expression analysis was performed using the limma package in R software (Version 4.5.1), which revealed that GADD45B had a log-fold change (logFC) of −0.6 with an adjusted *P*-value (adj.*P*.Val) < 0.01. A volcano plot was generated using the ggplot2 package (Fig. 1a) preliminarily confirmed that GADD45B was significantly downregulated in AIS. Gene Set Enrichment Analysis (GSEA) was performed using the clusterProfiler package (Fig. 1b) indicated that GADD45B is significantly associated with pathways including proteasome-mediated protein degradation, energy metabolism and mitochondrial function, genomic damage repair, and transcription-coupled nucleotide excision repair. The prediction results from RM2Target indicated that METTL14 may directly regulate GADD45B (Fig. 1c). RPISeq analysis suggested a high probability of interaction between GADD45B and BDNF, with a prediction probability of 0.65 using the Random Forest algorithm and 0.57 using the Support Vector Machine algorithm. Since both probabilities exceeded 0.5, these results collectively indicate a high likelihood of an interaction between GADD45B and BDNF.

**Fig. 2:**
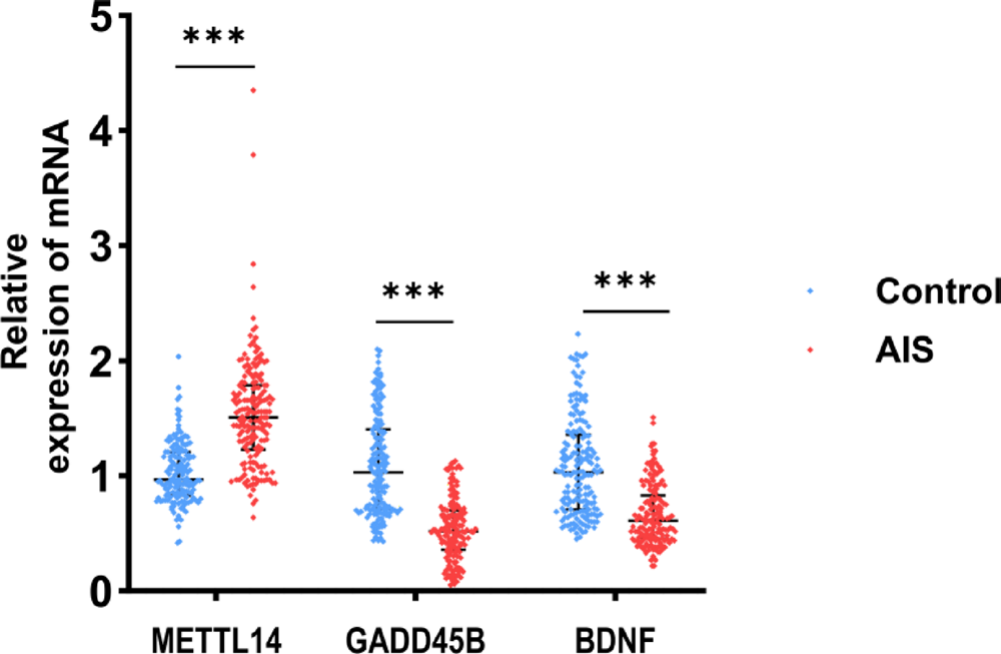
Relative expression levels of METTL14, GADD45B, and BDNF in the AIS group and Control group, *n* =177 (177 pairs of samples, technical replicates: *n* = 2). Statistical significance was defined as *P *< 0.05 (*P < 0.05, **P < 0.01, ***P < 0.001; ns: no statistical significance)

Subsequently, the Kolmogorov-Smirnov Test was performed on (differences in relative expression levels, METTL14/GADD45B/BDNF obtained (RT-qPCR, with results as follows: *d*: 0.32 (0.065,0.610), *D*_(177)=_0.072 (*P* = 0.026), (*Z* = −9.593, *P* < 0.001);*d*: −0.18 (−0.575,0.035), *D*_(177)_ = 0.088 (*P* = 0.002), (Z = −6.490, *P* < 0.001); *d*: −0.19 (−0.590,−0.040), *D*_(177)_ = 0.081 (*P* = 0.007), (Z=−6.757, *P* < 0.001). Compared with the Control group, the relative mRNA expression level of METTL14 was significantly increased in the AIS group, whereas those of GADD45B and BDNF were significantly decreased (Fig. 2). These results suggest that AIS may upregulate METTL14 gene expression, inhibit GADD45B expression under ischemic conditions, and downregulate BDNF expression. The primer sequences are shown in Table 3, and the proof of primer specificity obtained from NCBI is in Supplementary Material 1.

**Table 3:** Primer sequences

Gene	Primer sequences
METTL14	F GAACACAGAGCTTAAATCCCCA
	R TGTCAGCTAAACCTACATCCCTG
GADD45B	F TGCATACGAGAGACTTGGTTGA
	R ATGGGTACAGAGCAACTTCAG
BDNF	F TAACGGCGGCAGACAAAAAGA
	R TGCACTTGGTCTCGTAGAAGTAT
GAPDH	F ACAACTTTGGTATCGTGGAAGG
	R GCCATCACGCCACAGTTTC

### Correlation and Diagnostic Efficacy Analysis of METTL14/GADD45B/BDNF

**Fig. 3:**
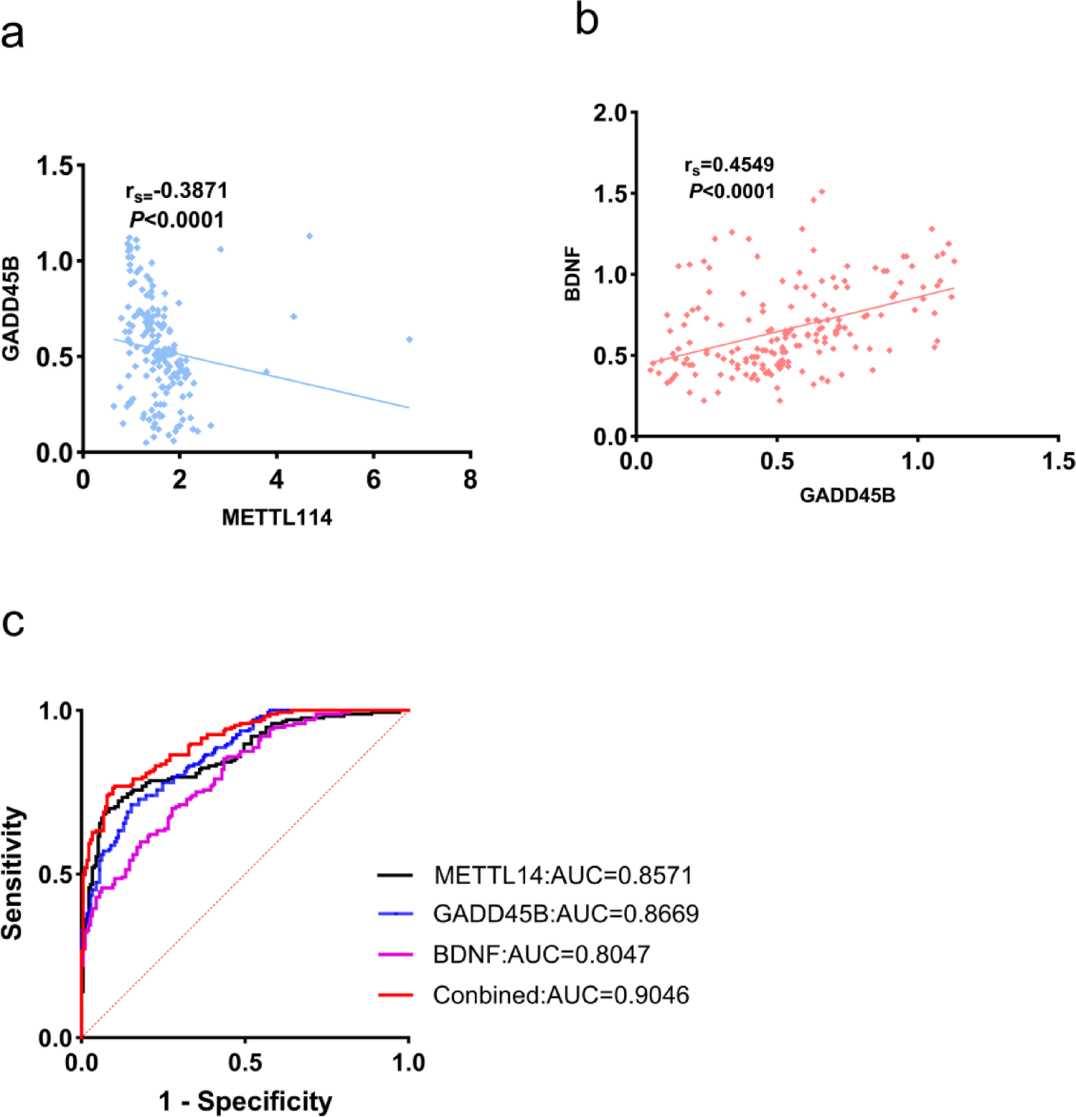
Correlation and diagnostic efficacy of METTL14/GADD45B/BDNF in AIS. **a** Correlation analysis between METTL14 and GADD45B. **b** Correlation analysis between GADD45B and BDNF. **c** Diagnostic efficacy of individual METTL14, GADD45B, BDNF, and their combined panel. Statistical significance was defined as *P* < 0.05 (*P < 0.05, **P < 0.01, ***P < 0.001, ns; ns: no statistical significance). *n* = 177 (177 pairs of samples, technical replicates: *n* = 2).

In the AIS group, the three genes were first subjected to normality analysis and analysis of variance, with the following results: METTL14 (*D*_(177)_ = 0.064, *P* = 0.070), GADD45B (*D*_(177)_ = 0.089, *P* = 0.002), and BDNF (*D*_(177)_ = 0.087, *P* = 0.002). The Levene’s Test for METTL14/GADD45B showed *F*_(1,352)_ = 14.112, *P* < 0.001, and for GADD45B/BDNF, it was *F*_(1,352)_ = 0.115, *P* = 0.735. The correlation analysis results for the three genes in the AIS group are shown in Fig. 3a (*r*_(352)_ = −0.3871, *P* < 0.0001). Spearman correlation analysis revealed a moderate negative correlation between METTL14 and GADD45B, while a moderate positive correlation was observed between GADD45B and BDNF (Fig. 3b) (*r*_(352)_ = 0.4549, *P* < 0.0001). Figure 3c shows the clinical diagnostic efficacy of these three target genes. For METTL14, the area under the curve (AUC) was 0.8571, with a 95% confidence interval (95% CI) of 0.8183–0.8959 (*P* < 0.0001); the cut-off value was 1.335, corresponding to a sensitivity of 68.93% and a specificity of 92.09%. For GADD45B, the AUC was 0.8669 (95% CI: 0.8314–0.9023, *P* < 0.0001), with a cut-off value of 0.6650, sensitivity of 71.19%, and specificity of 84.75%. For BDNF, the AUC was 0.8047 (95% CI: 0.7610–0.8484, *P* < 0.0001), with a cut-off value of 0.7550, sensitivity of 70.06%, and specificity of 72.32%. Notably, the combined panel of these three target genes exhibited higher diagnostic efficacy, with an AUC of 0.9046 (95% CI: 0.8751–0.9341, *P* < 0.0001), a sensitivity of 83.05%, and a specificity of 76.84%. Collectively, these results indicate that the combined use of these three target genes provides superior clinical diagnostic performance for AIS.

### Differences in METTL14/GADD45B/BDNF Expression Between Normal and OGD Conditions

**Fig. 4:**
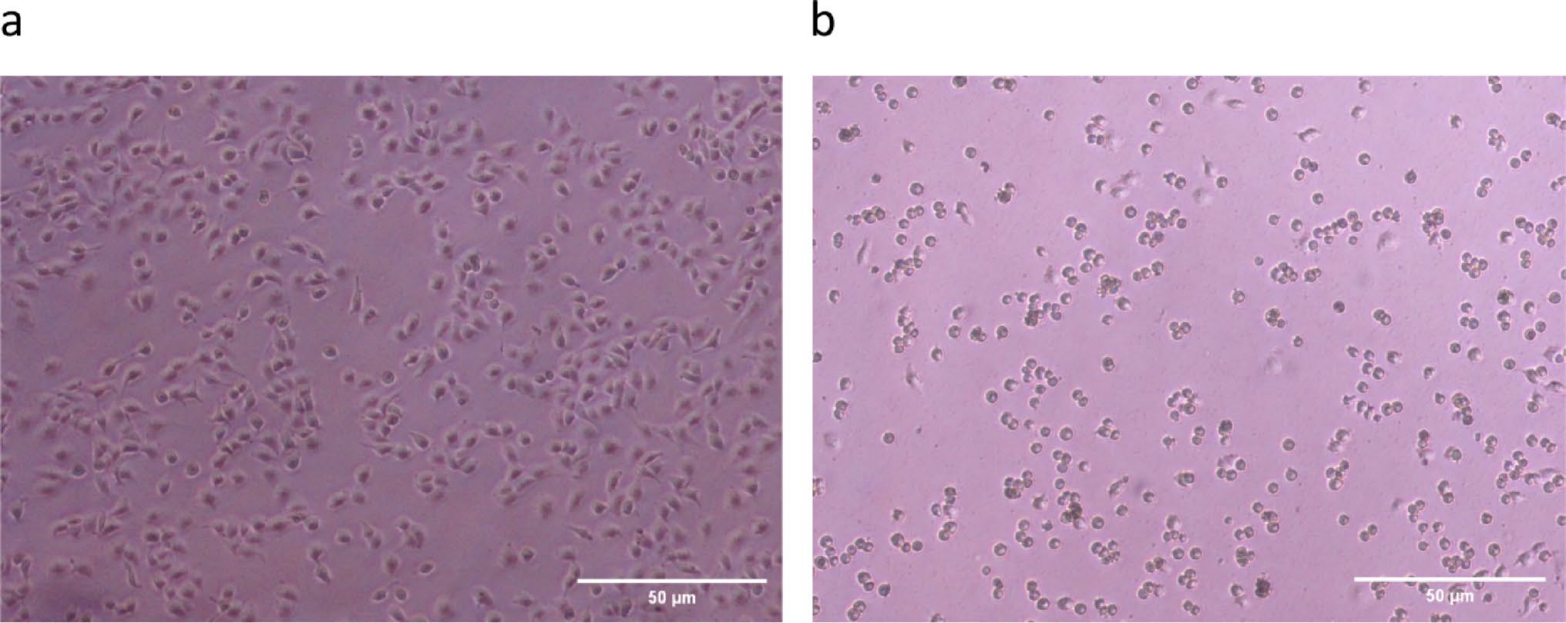
Changes in HUVECs morphology. **a** Normal HUVECs. **b** HUVECs after 6-hour OGD

**Fig. 5:**
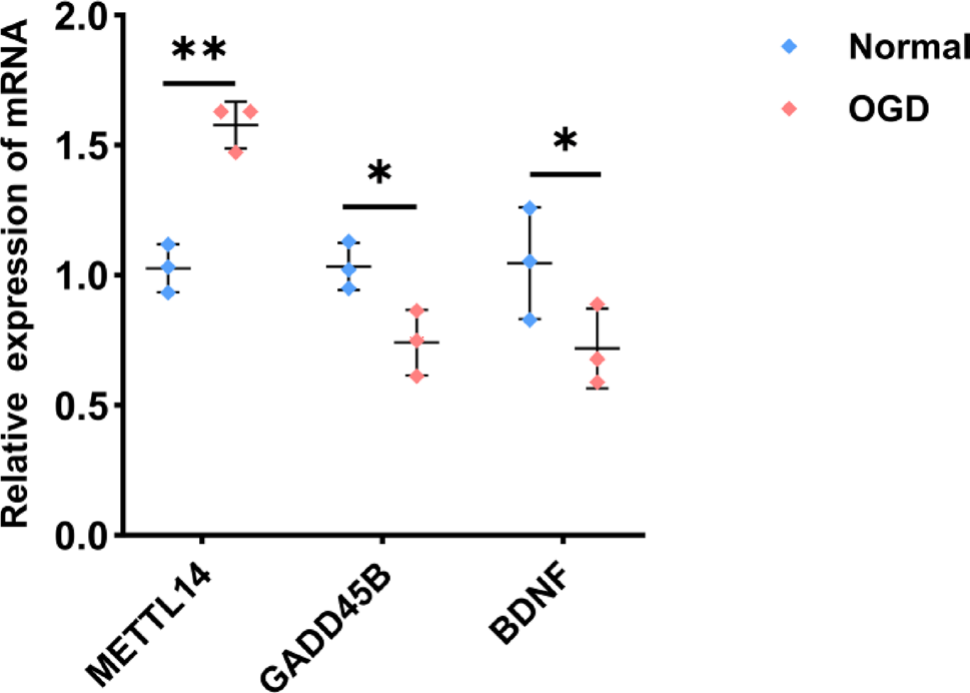
Expression of METTL14/GADD45B/BDNF in Normal and OGD group. Normality analysis: Normal group [METTL14 (*W*_(3)_ = 0.999, *P* = 0.940), GADD45B (*W*_(3)_ = 0.984, *P* = 0.756), BDNF (*W*_(3)_ = 0.999, *P* = 0.940)], OGD [METTL14 (*W*_(3)_ = 0.966, *P* = 0.647), GADD45B (*W*_(3)_ = 0.999, *P* = 0.927), BDNF (*W*_(3)_ = 0.995, *P* = 0.859)]. Homogeneity of variance test: [METTL14 (*F*_(1,4)_ = 0.047, *P* = 0.839), GADD45B (*F*_(1,4)_ = 0.191, *P* = 0.685), METTL14 (*F*_(1,4)_ = 0.179, *P* = 0.694)]. The results of differential analysis for METTL14/GADD45B/BDNF are as follows: *t *_(4)_ = 6.613 (*P* = 0.003), *t*_(4)_=−3.275 (*P* = 0.031), *t*_(4)_=−3.2119 (*P* = 0.033). A *P*-value < 0.05 was considered statistically significant (*P < 0.05, **P < 0.01, ***P < 0.001, ns; ns: indicates no statistically significant difference). Normal group = 3, OGD group = 3, technical replicates: *n* = 3

To further validate the regulatory relationship among METTL14, GADD45B, and BDNF, we established a HUVEC OGD model. The success of the OGD model was confirmed by CCK8 assay. As shown in Fig. 4a, normally cultured HUVECs exhibited a monolayer arrangement with a pebble-like dense structure, abundant cytoplasm, and well-defined boundaries. After 6 h of OGD treatment, the cell arrangement was significantly disrupted, with some cells shrinking and fragmenting, accompanied by a marked reduction in cell count and the appearance of cell debris (Fig. 4b). Table 4 demonstrated that compared to the Normal group, the survival rate of cells treated with OGD for 6 h was significantly reduced. RT-qPCR analysis revealed that the relative expression levels of METTL14 mRNA were significantly elevated, while those of GADD45B and BDNF genes were significantly decreased in the OGD group compared to the Normal group (Fig. 5). These findings suggest that OGD may enhance METTL14 gene expression, while GADD45B expression may be suppressed under ischemia-hypoxia conditions, and AIS may reduce BDNF expression.

**Table 4:** Analysis of cell viability using the CCK-8 assay for OGD 6h versus normal conditions

Groups	OD	*t*	*P*
Normal	1.109 ± 0.064	13.122	< 0.001
6 h OGD	0.598 ± 0.020		

Normality test: Normal group (*W*_(3)_=0.943, *P*=0.540), OGD group (*W*_(3)_=0.833, *P*=0.196); homogeneity of variance test (*F*_(1,4)_=3.913, *P*=0.119). Normal group = 3, OGD group=3, technical replicates: *n*=3. A *P*-value <0.05 was considered statistically significant (*P< 0.05, **P< 0.01, ***P< 0.001, ns:indicates no statistically significant difference)

### The Overall m^6^A RNA Content in Peripheral Blood and OGD HUVECs was Higher than that in the Control Group

**Fig. 6:**
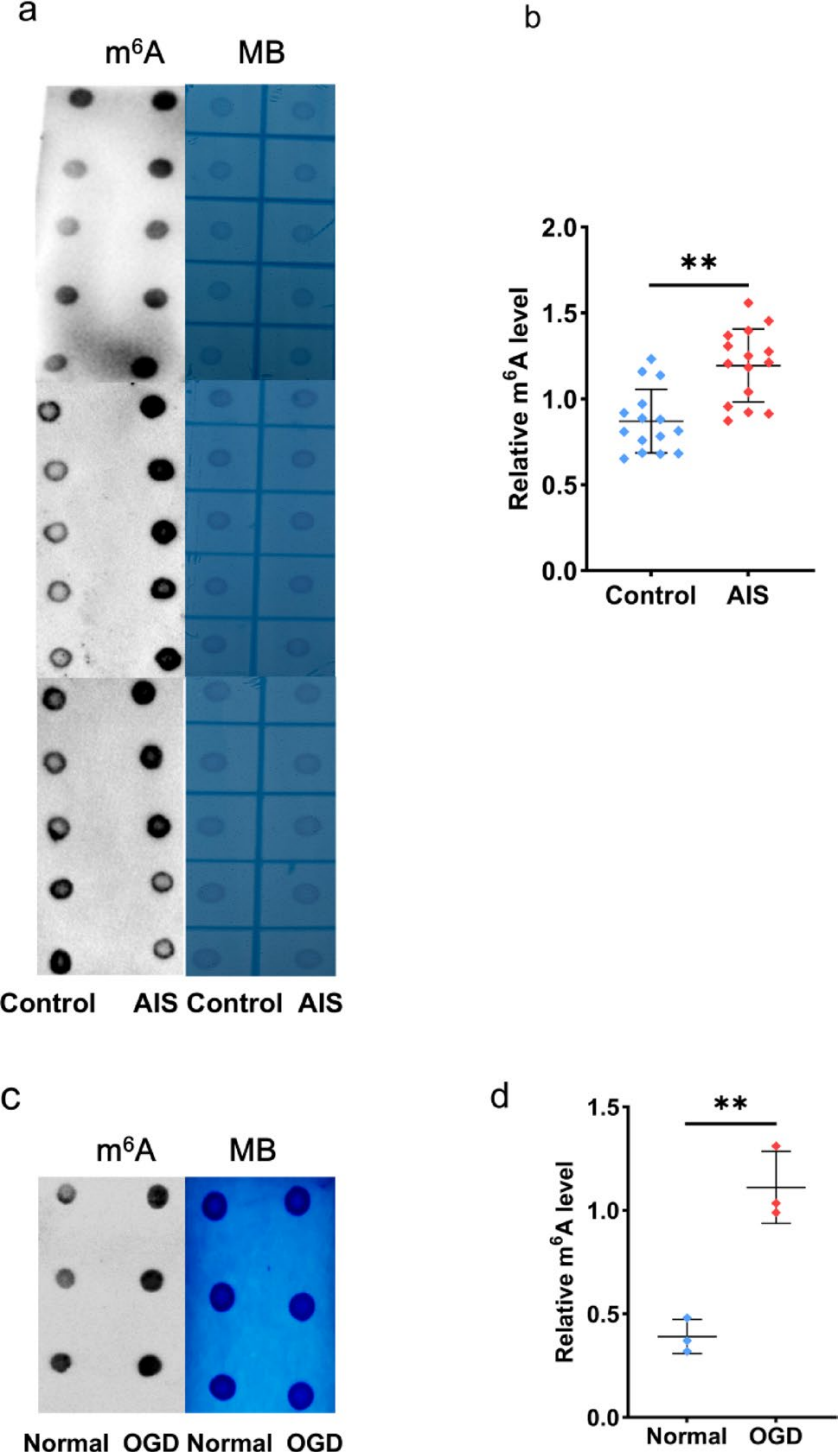
Dot blot experimental result diagram. **a**,** c** Dot blot development and methylene blue staining images; **b** Comparison of relative m^6^A levels in whole RNA between Control group and AIS group (15 pairs of samples, technical replicates: *n* = 2), normality analysis of difference values (*W*_(15)_=0.862, *P* = 0.025), using the Kruskal-Wallis test (Z=−2.953, *P* = 0.003)。 **d** Comparison of relative m^6^A levels in whole RNA between Normal group and OGD group (Normal group = 3, OGD group = 3, technical replicates: *n* = 3). Normality analysis: Normal group (*W*_(3)_ = 0.958, *P* = 0.604), OGD group (*W*_(3)_ = 0.853, *P* = 0.250), homogeneity of variance test (*F*_(1,4)_ = 3.028, *P* = 0.157), using paired sample t-test(*t*_(4)_ = 6.480,*P* = 0.003). A *P*-value < 0.05 was considered statistically significant (*P < 0.05, **P < 0.01, ***P < 0.001; ns indicates no statistical difference)

The Dot blot experiment was performed to verify the relative differences in m^6^A RNA levels between peripheral blood of AIS patients, OGD HUVECs, and the Control Normal group. As shown in Fig. 6a, the Dot blot images of blood samples and methylene blue staining are presented. Figure 6b demonstrates that the total m^6^A RNA content in the AIS group was significantly higher than that in the Control group, with statistical significance. Figure 6c displays the Dot blot images and methylene blue staining of OGD HUVECs and the Normal group. Figure 6d indicates that the total m^6^A RNA content in OGD HUVECs was significantly higher than that in the Normal group, suggesting that the occurrence of AIS may increase m^6^A RNA levels. The original Dot blot images and replicate experiments are provided in Supplementary Material 2. The proof of the specificity of the primary antibody is presented in Supplementary Material 3.

### METTL14 May Reduce the Stability of GADD45B mRNA, Thereby Indirectly Leading to Decreased BDNF Expression

**Fig. 7:**
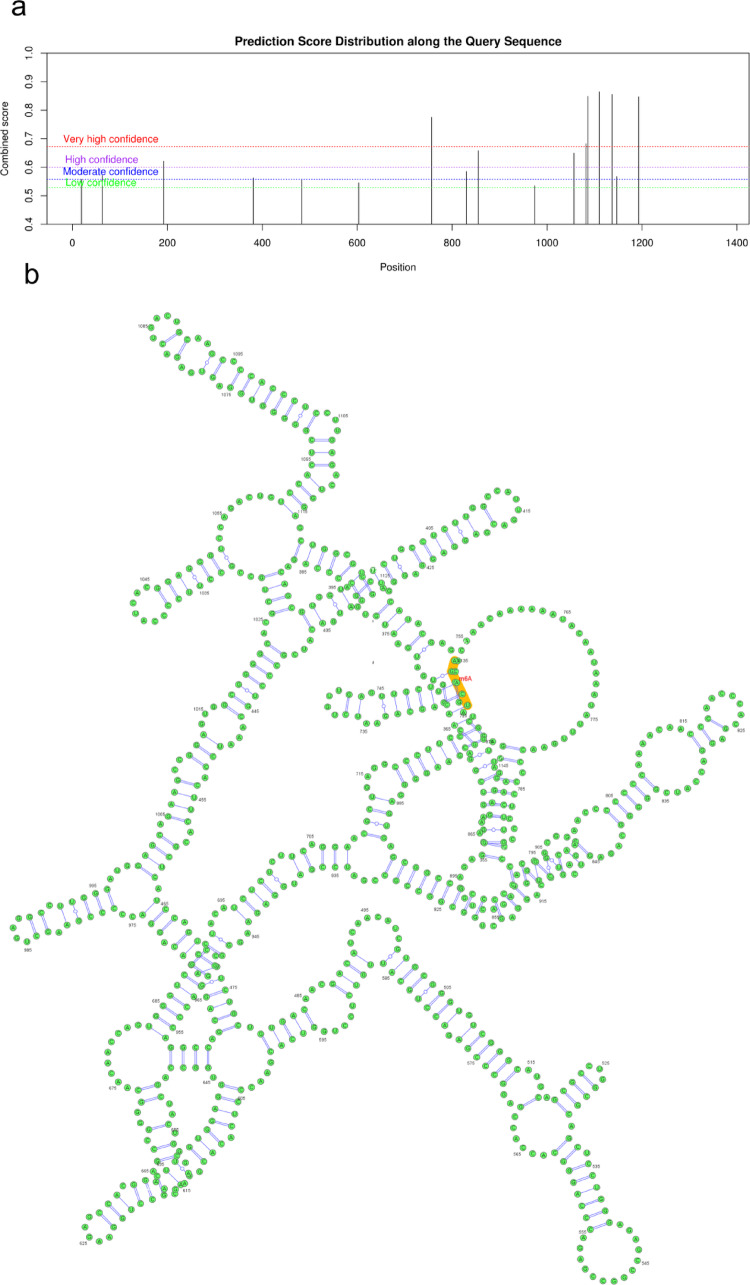
Prediction of GADD45B m^6^A modification sites. **a** Prediction of m^6^A modification sites in GADD45B mRNA. **b** Secondary structure diagram of GADD45B mRNA and related m^6^A sites

METTL14 is a well-characterized m^6^A methyltransferase. Spot blot experiments confirmed that the overall m⁶A RNA levels in the AIS group and OGD group were higher than those in the Control group and Normal group. RM2Target predictions indicated that METTL14 directly regulates GADD45B. Additionally, RT-qPCR experiments demonstrated that METTL14 was significantly upregulated in both the AIS group and OGD group, with a negative correlation between METTL14 and GADD45B in the AIS group, and METTL14 was also significantly upregulated in the OGD group. Consequently, researchers used SRAMP to predict the m⁶A modification sites of GADD45B. As shown in Fig. 7a, GADD45B has multiple potential m^6^A modification sites, some of which are high-confidence sites. Based on the prediction results, the 1000–1200 bp region was identified as the primary modification fragment. Figure.7b illustrates the secondary structure of GADD45B mRNA.

**Fig. 8:**
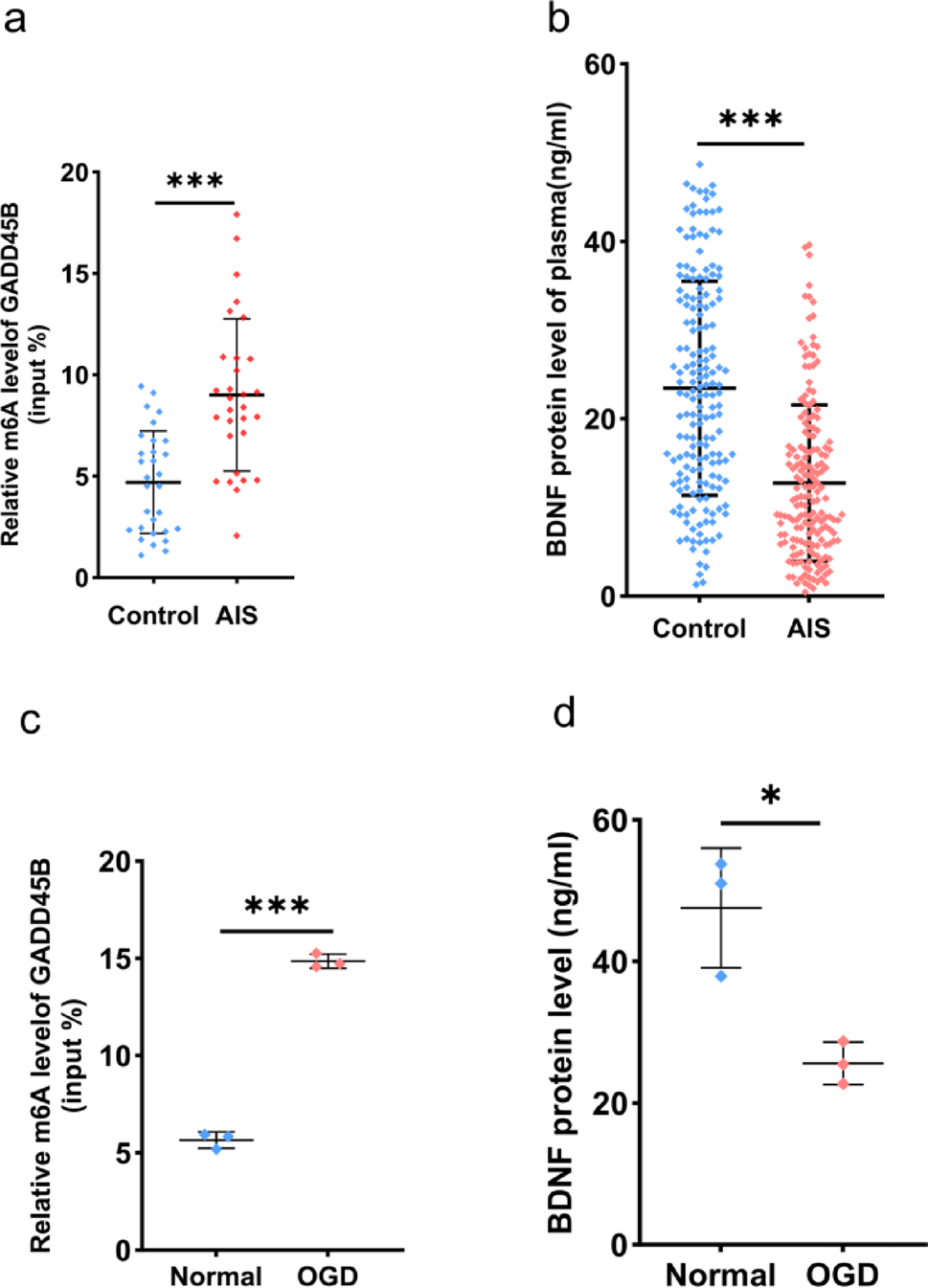
MeRIP-RT-qPCR and ELISA plots. **a** Comparison of relative expression levels of GADD45B m^6^A mRNA between the AIS group and the Control group, with normality analysis of the difference (*W*_(30)_=0.942, *P* = 0.105), *n* = 30 (30 paired samples, technical replicates: *n* = 2). **b** Comparison of plasma BDNF protein levels between the AIS group and the Control group, with normality analysis of the difference (*W*_(177)_ = 0.052, *P* = 0.200), *n* = 177 (177 paired samples, technical replicates: *n* = 2). **c** Comparison of relative expression levels of GADD45B m^6^A mRNA between the Normal group and the OGD group. Normality analysis: Normal group (*W*_(3)_ = 0.836, *P* = 0.203), OGD group (*W*_(3)_ = 0.910, *P* = 0.418); homogeneity of variance test: (*F*_(1,4)_ = 0.129, *P* = 0.737), *n* = 3 (Normal group = 3, OGD group = 3, technical replicates: *n* = 3). **d** Comparison of BDNF protein levels between the Normal group and the OGD group, with normality analysis: Normal group (*W*_(3)_ = 0.877, *P* = 0.315), OGD group (*W*_(3)_ = 0.998, *P* = 0.919); homogeneity of variance test: (*F*_(1,4)_ = 4.660, *P* = 0.097), independent samples *t*-test (*t*_(4)_ = 4.423, * P*= 0.013), (Normal group = 3, OGD group = 3, technical replicates: *n* = 3). Statistical significance was defined as *P * < 0.05 (*P < 0.05, **P < 0.01, ***P < 0.001; ns: no statistical significance)

The relative m^6^A mRNA content of GADD45B in each group obtained by MeRIP-RT-qPCR. The analysis results showed that compared with the Control group or Normal group, the relative m^6^A modification level of GADD45B mRNA in the AIS group or OGD group was significantly increased (Fig. 8a, c), which indicated that AIS might increase its m^6^A modification level.

RT-qPCR experiments have confirmed that GADD45B and BDNF are significantly down-regulated in the AIS and OGD groups. Further ELISA experiments have confirmed that BDNF is also significantly down-regulated in the AIS and OGD groups (Fig. 8b, d). As mentioned earlier, there is a positive correlation between GADD45B and BDNF, and the above RPISeq results also indicate that there is a high possibility of interaction between GADD45B and BDNF.

**Fig. 9:**
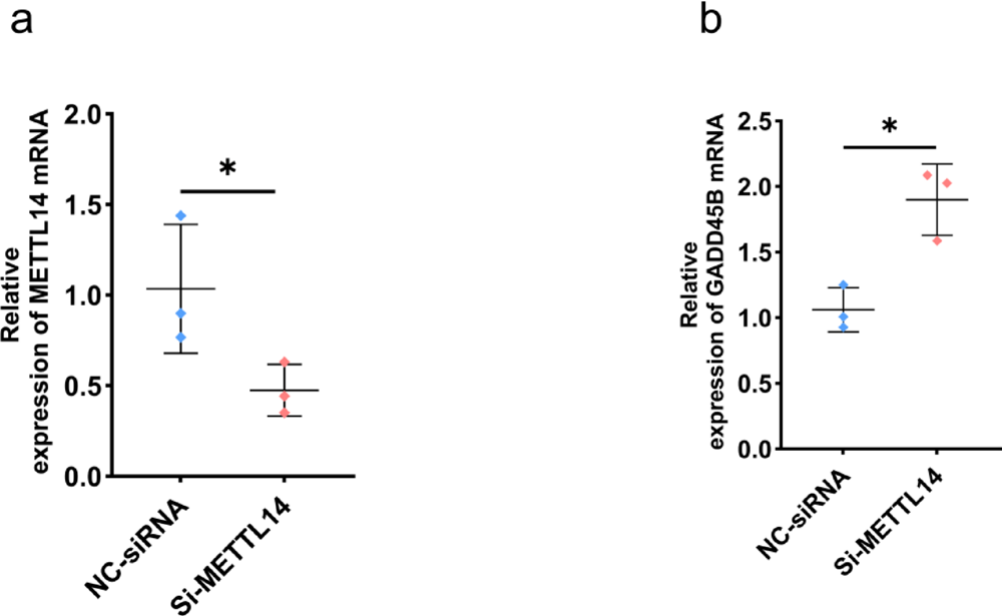
The expression levels of METTL14/GADD45B in HUVECs after 6 h of OGD following transfection. **a** METTL14 expression in the Si-METTL14 group, normality analysis: NC-siRNA group (*W*_(3)_ = 0.890, *P* = 0.354), Si-METTL14 group (*W*_(3)_ = 0.987, *P* = 0.784), homogeneity of variance test: (*F*_(1,4)_ = 6.273, *P* = 0.066), independent Samples t-test(*t*_(4)_=−2.820, *P* = 0.048), (NC-siRNA group  = 3, Si-METTL14 group  = 3, technical replicates: *n* = 3). **b** GADD45B expression in NC-siRNA and Si-METTL14 groups, normality analysis: NC-siRNA group (*W*_(3)_ = 0.921, *P* = 0.456), Si-METTL14 group (*W*_(3)_ = 0.840, *P* = 0.215), homogeneity of variance test: (*F*_(1,4)_ = 1.481, *P* = 0.290), independent Samples t-test(*t*_(4)_ = 4.528, *P* = 0.011), (NC-siRNA group  = 3, Si-METTL14 group  = 3, technical replicates: *n* = 3,technical replicates: *n* = 3). Statistical significance was defined as *P* < 0.05 (*P < 0.05, **P < 0.01, ***P < 0.001; ns: no statistical significance)

**Fig. 10:**
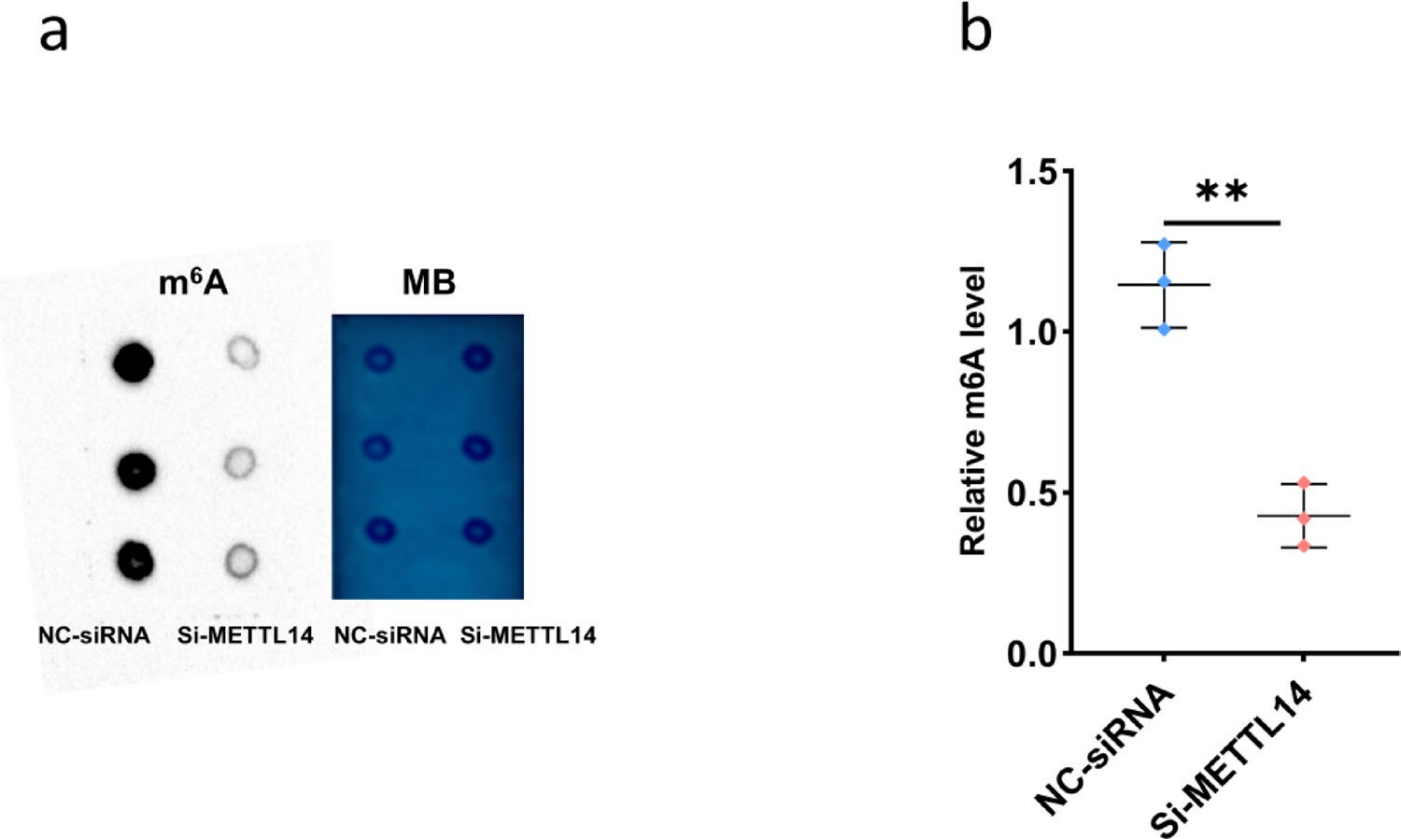
The Dot blot results of OGD HUVECs at 6 h post-transfection were observed. **a** Development and methylene blue staining images. **b** Comparison of m^6^A relative levels of total RNA between NC-siRNA and Si-METTL14 groups. Normality analysis: NC-siRNA group (*W*_(3)_ = 0.994, *P* = 0.847); Si-METTL14 group (*W*_(3)_ = 0.995, *P* = 0.869). Homogeneity of variance test: (*F*_(1,4)_ = 0.216, *P* = 0.666), independent Samples t-test (*t*_(4)_ = 7.507, *P* = 0.002), (NC-siRNA group = 3, Si-METTL14 group  = 3, technical replicates: *n* = 3). Statistical significance was defined as *P* < 0.05 (*P < 0.05, **P < 0.01, ***P < 0.001; ns: no statistical significance)

**Fig. 11:**
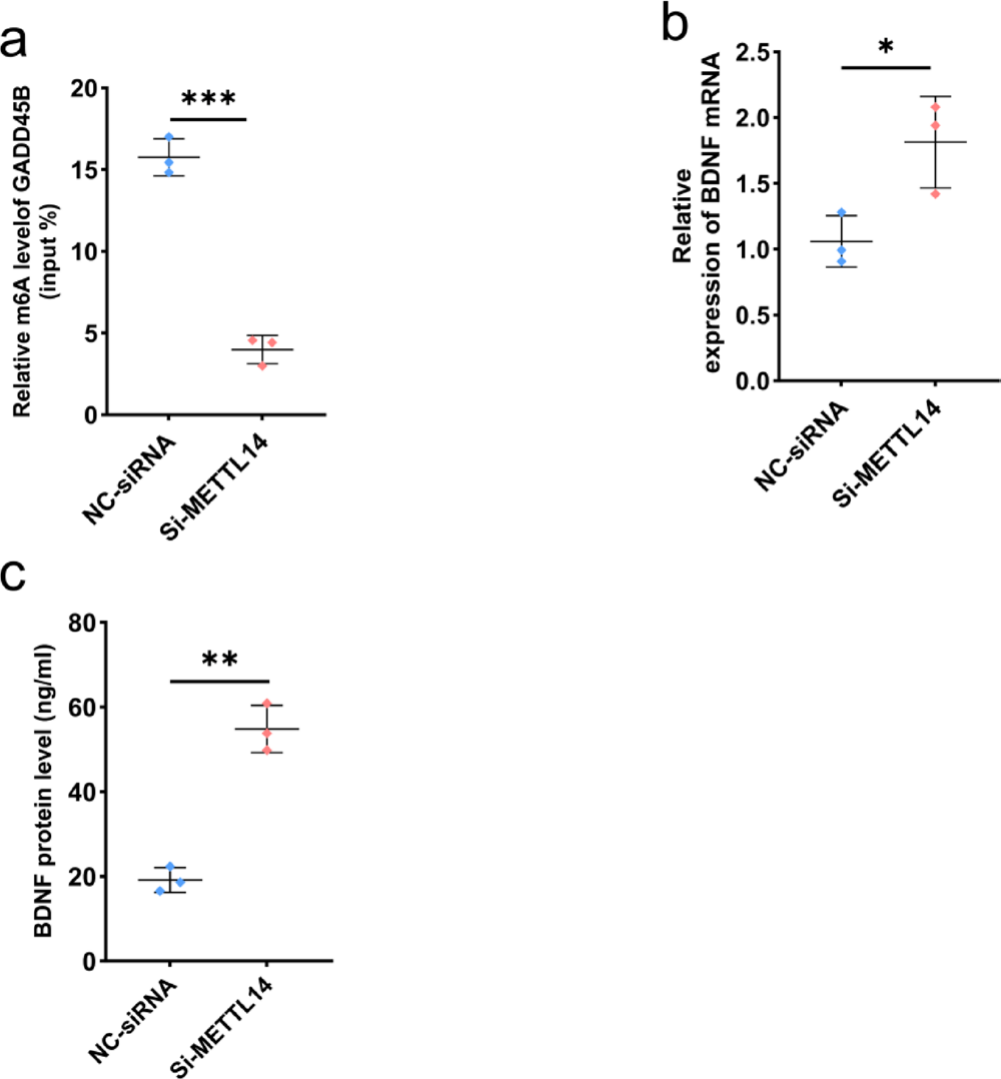
Expression of GADD45B m^6^A mRNA and BDNF in HUVECs at 6 h after transfection with OGD. **a** Comparison of relative GADD45B m^6^A mRNA expression levels between NC-siRNA and Si-METTL14 groups, normality analysis: NC-siRNA group (*W*_(3)_ = 0.814, *P* = 0.847), Si-METTL14 group (*W*_(3)_ = 0.216, *P* = 0.148); homogeneity of variance test: (*F*_(1,4)_ = 0.288, *P* = 0.620) (NC-siRNA group  = 3, Si-METTL14 group  = 3, technical replicates: *n* = 3). **b** BDNF mRNA expression in NC-siRNA and Si-METTL14 groups, normality analysis: NC-siRNA group (*W*_(3)_ = 0.912, *P* = 0.425), Si-METTL14 group (*W*_(3)_= 0.901, *P *= 0.388); homogeneity of variance test: (*F*_(1,4)_ = 1.681, *P* = 0.265), independent Samples t-test (*t*_(4)_ = 3.273, *P* = 0.031), (NC-siRNA group = 3, Si-METTL14 group = 3, technical replicates: *n* = 3). **c** Comparison of plasma BDNF protein levels between NC-siRNA and Si-METTL14 groups. Normality analysis: NC-siRNA group (W_(3)_ = 0.973, *P* = 0.684) and Si-METTL14 group (W_(3)_ = 0.965, *P* = 0.642). Variance homogeneity test: (*F*_(1,4)_ = 3.748, *P* = 0.125), independent Samples t-test (*t*_(4)_ = 5.048, *P* = 0.007), (NC-siRNA group = 3, Si-METTL14 group = 3, technical replicates: *n* = 3). Statistical significance was defined as *P* < 0.05 (*P < 0.05, **P < 0.01, ***P < 0.001; ns: no statistical significance)

To validate the regulatory relationship between METTL14 and GADD45B, we constructed a HUVEC OGD model and siRNA, with transfection efficiency confirmed by RT-qPCR. As shown in Fig. 9a, the Si METTL14 group exhibited decreased METTL14 expression compared to the negative Control group (RT-qPCR), indicating successful transfection. Concurrently, GADD45B expression was upregulated (Fig. 9b). Dot blot results demonstrated a reduction in overall m^6^A RNA content (Fig. 10a, b). MeRIP-RT-qPCR revealed decreased levels of GADD45B m^6^A mRNA (Fig. 11a). RT-qPCR and ELISA results indicated increased BDNF expression (Fig. 11b, c).

**Fig. 12:**
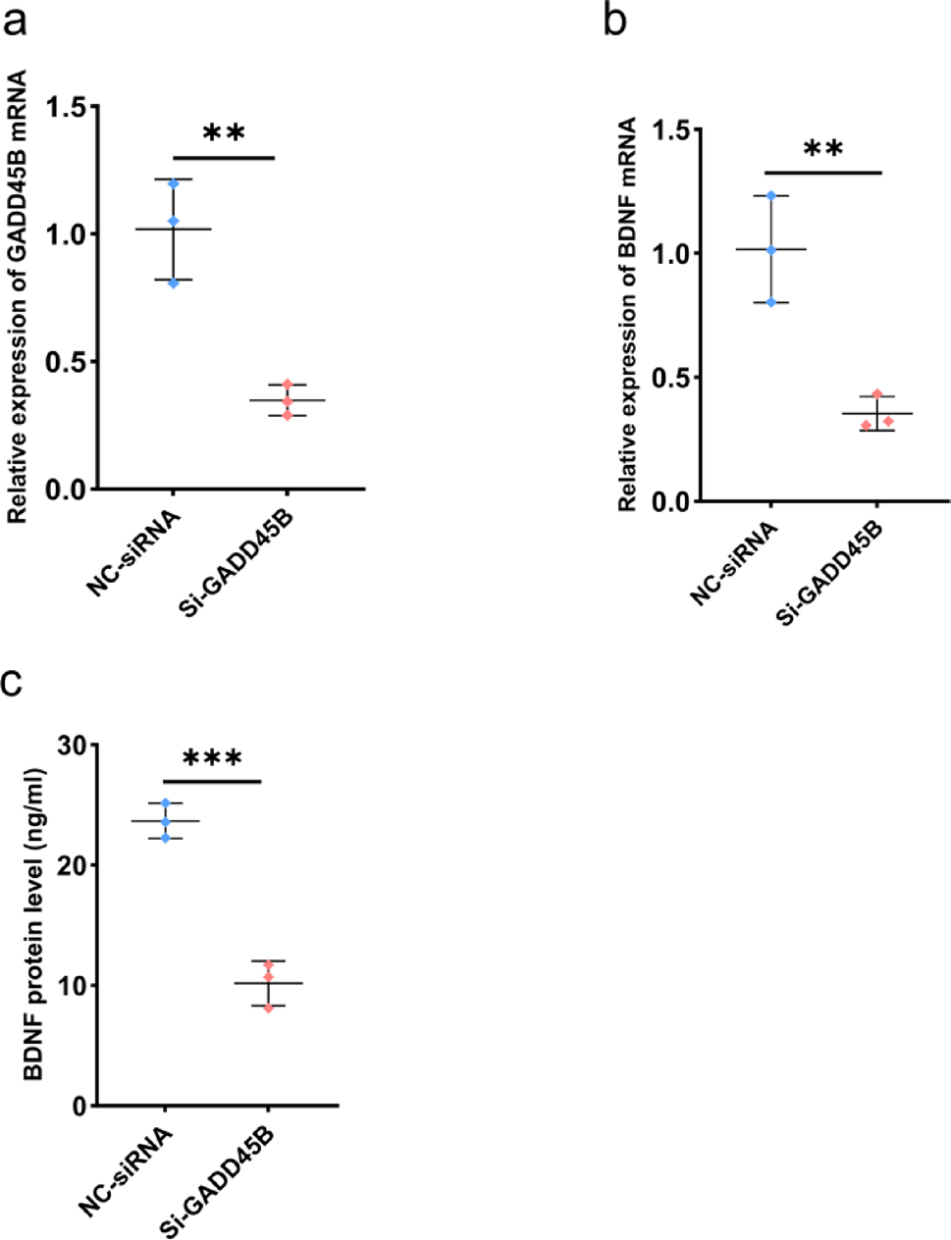
Expression of GADD45B mRNA and BDNF in HUVECs treated with 6hOGD after transfection. **a** Comparison of relative GADD45B mRNA expression levels between NC-siRNA and Si-GADD45B groups (NC-siRNA  group = 3, Si-GADD45B group  = 3, technical replicates: *n* = 3). Normality analysis: NC-siRNA group (*W*_(3)_ = 0.980, *P*= 0.729; Si-METTL14 group (*W*_(3)_ = 0.996, *P* = 0.878); homogeneity of variance test: (*F*_(1,4)_ = 2.919, *P* = 0.163) independent Samples t-test (*t*_(4)_=−5.635, *P* = 0.005). **b** BDNF mRNA expression in NC-siRNA and Si-GADD45B groups (NC-siRNA group = 3, Si-GADD45B group  = 3, technical replicates: *n* = 3). Normality analysis: NC-siRNA group (*W*_(3)_ = 1.000, *P* = 0.974; Si-METTL14 group (*W*_(3)_ = 0.847, *P* = 0.232); homogeneity of variance test: (*F*_(1,4)_ = 1.630, *P* = 0.271), independent Samples t-test (*t*_(4)_=−5.080, *p* = 0.007). **c** BDNF protein levels between NC-siRNA and Si-GADD45B groups (NC-siRNA group  = 3, Si-GADD45B group  = 3, technical replicates: *n* = 3). Normality analysis: NC-siRNA group (*W*_(3)_ = 0.998, *P* = 0.913; Si-METTL14 group (*W*_(3)_ = 0.936, *P* = 0.513); homogeneity of variance test: (*F*_(1,4)_ = 0.365, *P* = 0.578). Statistical significance was defined as *P* < 0.05 (*P < 0.05, **P < 0.01, ***P < 0.001; ns: no statistical significance)

To investigate the regulatory relationship between GADD45B and BDNF expression, we knocked down GADD45B, and the transfection efficiency was validated by qRT-PCR. As shown in Fig. 12a, the Si GADD45B group exhibited a significant decrease in GADD45B expression compared to the negative Control group, indicating successful transfection. RT-qPCR and ELISA results demonstrated a decrease in BDNF expression (Fig. 12b, c).

In conclusion, based on bioinformatics analysis, RT-qPCR and MeRIP-RT-qPCR results, as well as ELISA data, it can be inferred that the upregulation of METTL14 in AIS leads to an increase in the m^6^A methylation level of GADD45B mRNA. The m^6^A modification reduces the stability of GADD45B mRNA, which indirectly results in decreased BDNF expression.

### Logistic Regression Analysis and Nomogram Model for AIS

#### Univariate Logistic Regression Analysis for AIS

After excluding smoking, alcohol consumption, and triglyceride (TG) factors (Table 2), univariate logistic regression analysis was performed to evaluate the correlation between biochemical indicators and categorical variables (e.g., hypertension and diabetes mellitus) and AIS. For METTL14, GADD45B, and BDNF, ΔCT values (×10) obtained from RT-qPCR were used for logistic regression analysis (Mes et al. 2017; Zhao et al. 2017).

As shown in Table 5, all factors except TC exhibited statistically significant correlations except for total cholesterol (TC). The detailed results of the univariate logistic regression analysis were as follows: total protein (TP): *OR* = 0.872, 95% CI: 0.832–0.913, *P* < 0.001; albumin (ALB): *OR* = 0.730, 95% CI: 0.660–0.808, *P* < 0.001; globulin (GLOB): *OR* = 1.174, 95% CI: 1.097–1.257, *P* < 0.001; glucose (GLU): *OR* = 1.510, 95% CI: 1.284–1.775, *P* < 0.001; TC: *OR* = 0.965, 95% CI: 0.858–1.086, *P* = 0.558; high-density lipoprotein cholesterol (HDL-C): *OR* = 0.324, 95% CI: 0.163–0.455, *P* = 0.001; low-density lipoprotein cholesterol (LDL-C): *OR* = 1.508, 95% CI: 1.137–2.001, *P* = 0.004; diabetes: *c* = 4.875, 95% CI: 2.847–8.348, *P* < 0.001; hypertension: *OR* = 10.714, 95% CI: 4.938–23.247, *P* < 0.001; dyslipidemia: *OR* = 1.643, 95% CI: 1.027–2.628, *P* = 0.038; METTL14: *OR* = 0.275, 95% CI: 0.159–0.475, *P* < 0.001; GADD45B: *OR* = 1.360, 95% CI: 1.237–1.495, *P* < 0.001; BDNF: *OR* = 2.851, 95% CI: 1.911–4.252, *P* < 0.001; BDNF protein: *OR* = 0.900, 95% CI: 0.871–0.930, *P* < 0.001. These results indicate that TP, ALB, HDL-C were protective factors against AIS, while GLOB, GLU, LDL-C, hypertension, diabetes mellitus, and dyslipidemia were risk factors for AIS. Given that a smaller ΔCT value corresponds to a higher relative expression level, METTL14 was identified as a risk factor for AIS, while GADD45B and BDNF were regarded as protective factors for AIS.

**Table 5:** Univariate logistic regression analysis of AIS

Variable	*β*	SE	Waldχ^2^	*P*	*OR*	95%CI
TP	−0.137	0.024	33.266	< 0.001^***^	0.872	0.832–0.913
ALB	−0.314	0.051	37.369	< 0.001^***^	0.730	0.660–0.808
GLOB	0.160	0.035	21.252	< 0.001^***^	1.174	1.097–1.257
GLU	0.412	0.083	24.812	< 0.001^***^	1.510	1.284–1.775
TC	−0.035	0.060	0.344	0.558	0.965	0.858–1.086
HDL-C	−1.126	0.352	10,226	0.001^**^	0.324	0.163–0.455
LDL-C	−0.411	0.144	8.117	0.004^**^	1.508	1.137–2.001
Diabetes	1.584	0.274	33.317	< 0.001^***^	4.875	2.847–8.348
Hypertension	2.372	0.395	36.010	< 0.001^***^	10.714	4.938–23.247
Dyslipidemia	0.496	0.240	4.290	0.038^*^	1.643	1.027–2.628
METTL14	−1.291	0.279	21.349	< 0.001^***^	0.275	0.159–0.475
GADD45B	0.308	0.048	40.553	< 0.001^***^	1.360	1.237–1.495
BDNF	1.048	0.204	26.379	< 0.001^***^	2.851	1.911–4.252
BDNF Protein	−0.105	0.017	40.652	< 0.001^***^	0.900	0.871–0.930

*β* regression coefficient; *SE* standard error; *Waldχ*^2^ wald square test statistic; *OR* odds ratio; *CI* confidence interval; *TP* total protein; *ALB* albumin; *GLOB* globulin; *GLU* glucose; *TC* total cholesterol; *HDL-C* high-density lipoprotein cholesterol; *LDL-C* low-density lipoprotein cholesterol; *METTL14* methyltransferase-like protein 14, *GADD45B* growth arrest and DNA damage-inducible protein 45 beta, *BDNF* brain-derived neurotrophic factor; Statistical significance was defined as *P*<0.05 (*P <0.05, **P < 0.01, ***P < 0.001; ns: no statistical significance). BDNF Protein: BDNF protein levels were measured using ELISA. 177 (177 pairs of samples)

#### Multivariate Logistic Regression Analysis of AIS

As shown in Table 5, all factors except TC were statistically significant in the correlation analysis; forward stepwise multivariate logistic regression analysis was performed on these significant factors, and Table 6 shows that the ΔCT (×10) values of METTL14/GADD45B/BDNF, GLOB, HDL-C, Hypertension, Diabetes, and BDNF Protein were included in the multivariate model, with a likelihood ratio test result of *P* < 0.001.

**Table 6:** Multivariate logistic regression analysis of AIS

Variable	*β*	SE	Waldχ^2^/Score	*P*	*OR*	95%CI
TP	–	–	0.046	0.831	–	–
ALB	–	–	0.043	0.835	–	–
GLOB	0.310	0.062	25.156	< 0.001^***^	1.364	1.208–1.539
GLU	–	–	0.380	0.537	–	–
HDL-C	−0.269	0.047	32.404	< 0.001^***^	0.764	0.697–0.838
LDL-C	–	–	0.522	0.470	–	–
Hypertension	2.151	0.553	15.143	< 0.001^***^	8.593	2.908–25.389
Diabetes	1.219	0.385	10.007	0.002^**^	3.382	1.590–7.197
Dyslipidemia	–	–	0.162	0.687	–	–
METTL14	−1.258	0.549	5.251	0.022^*^	0.284	0.097–0.834
GADD45B	0.054	0.016	11.451	< 0.001^***^	1.055	1.023–1.089
BDNF	1.303	0.498	6.849	0.009^**^	3.680	1.387–9.763
BDNF Protein	−0.108	0.021	26.715	< 0.001^***^	0.898	0.862–0.935

*β* regression coefficient, *SE* standard error; *Waldχ*^2^ wald square test statistic; Score test statistic; *OR* odds ratio; *CI* confidence interval; *TP* total protein; *ALB* albumin; *GLOB* globulin; *GLU* glucose; *TC* total cholesterol; *HDL-C* high-density lipoprotein cholesterol; *LDL-C* low-density lipoprotein cholesterol; *METTL14* methyltransferase-like protein 14; *GADD45B* growth arrest and DNA damage-inducible protein 45 beta; *BDNF* brain-derived neurotrophic factor; Statistical significance was defined as *P* < 0.05 (*P < 0.05, **P < 0.01, ***P < 0.001; ns: not statistically significant). BDNF Protein: BDNF protein levels were measured using ELISA. 177 (177 pairs of samples).

### Construction and Validation of Nomogram for AIS Risk Prediction Model

**Fig. 13:**
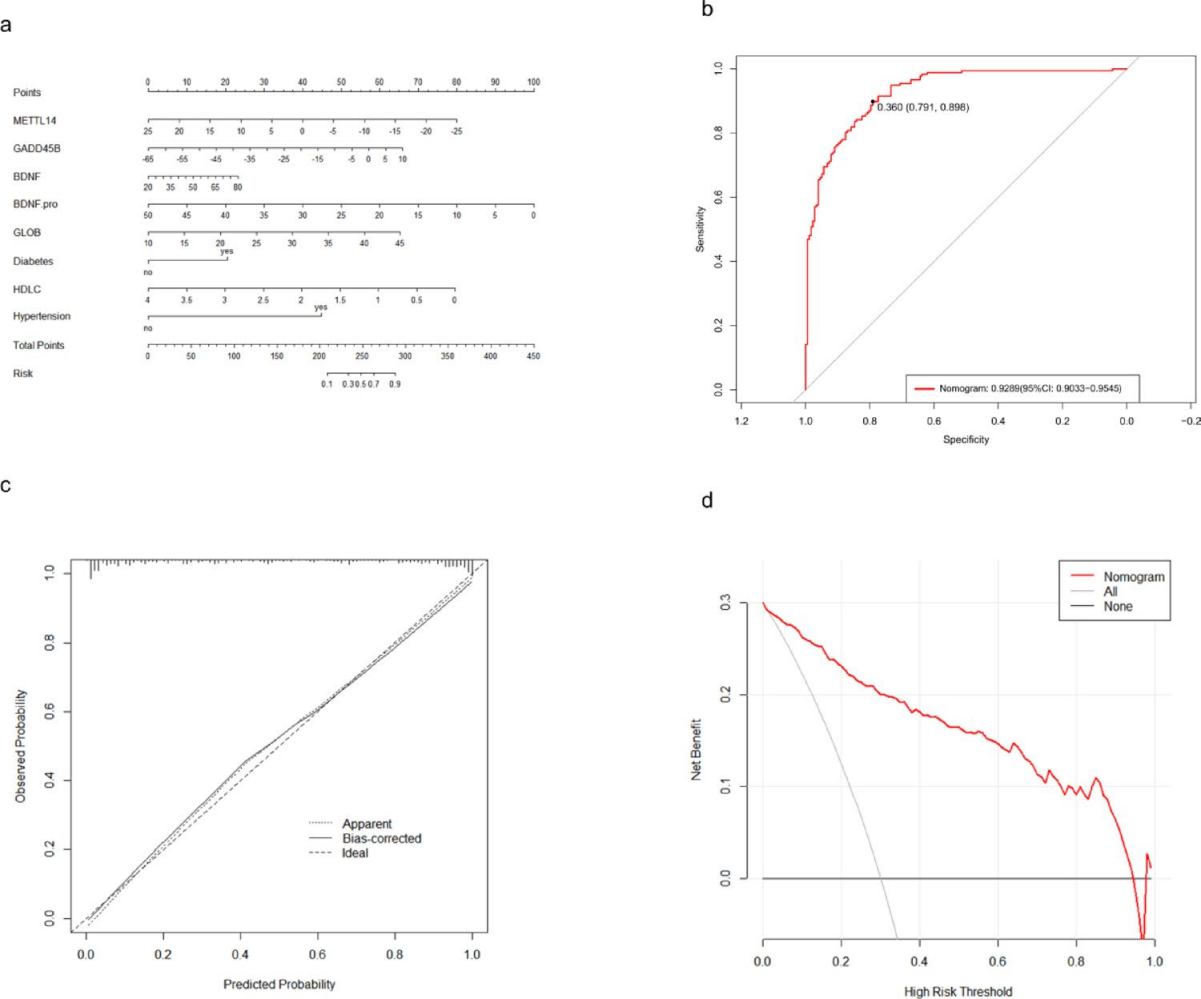
Construction and validation of nomogram for AIS risk prediction model. **a** Nomogram. **b** ROC curve. **c** Calibration curve. **d** DCA model. *n* = 177 (177 pairs of samples)

A nomogram was constructed using the factors included in the multivariate logistic regression model (Fig. 13a), aiming to assist clinicians in predicting the risk of AIS in patients or healthy individuals. This tool assigns scores to each influencing factor, allowing the calculation of individual AIS risk.The ROC curve showed that the nomogram had strong discriminative ability, with an AUC of 0.9289 (95%CI: 0.9033–0.9545) (Fig. 13b), cutoff value of 0.360, sensitivity of 0.791, and specificity of 0.898. Additionally, the calibration curve demonstrated strong consistency between the predicted results of the nomogram and the actual observations, verifying its accuracy (Fig. 13c). Decision curve analysis (DCA) was performed to evaluate the clinical utility of the nomogram. As shown in Fig. 13d, compared with the traditional model, the clinical model incorporating ΔCT (10×) of METTL14, GADD45B, BDNF, GLOB, HDL-C, hypertension, diabetes, and BDNF protein exhibited superior net benefits across a wide range of threshold probabilities.

## Discussion

AIS is a neurological disorder characterized by a high risk of morbidity and a poor prognosis. Currently, there are significant limitations to both therapeutic strategies and early diagnostic approaches for this condition. Therefore, there is an urgent need to enhance the predictive efficacy and optimize treatment regimens for AIS. The samples in this study are human peripheral blood and HUVECs. RT-qPCR experiments demonstrated that the expression levels of METTL14, GADD45B, and BDNF in the peripheral blood of AIS and OGD HUVECs are different from those in the Control group or Normal group. Among them, METTL14 was upregulated, while GADD45B and BDNF were downregulated. ELISA experiments also confirmed that BDNF is downregulated in AIS. Correlation analysis indicated a negative correlation between METTL14 and GADD45B, and a positive correlation between GADD45B and BDNF. ROC analysis further demonstrated that the diagnostic efficacy of METTL14, GADD45B, and BDNF was superior to that of the traditional influencing factors. Moreover, dot blot assays along with methylated RNA immunoprecipitation-quantitative polymerase chain reaction (MeRIP-RT-qPCR) experiments showed that the m^6^A RNA modification level of GADD45B in patients with AIS was elevated compared to that in the Control group. In conclusion, these findings underscore the significant role of the METTL14/GADD45B m^6^A methylation/BDNF axis in AIS and highlight its potential diagnostic value. Peripheral blood is among the most frequently used specimens in clinical testing and is commonly used for the diagnosis and monitoring of infectious diseases, malignant tumors, hematological disorders, and various other medical conditions (Zhang et al. 2023; Brown et al. 2019). Peripheral blood collection has several distinct advantages, including minimal invasiveness, brief collection duration, and high efficiency (Guo et al. 2022). Fan et al. (2025) demonstrated that plasma fat mass and the concentration of obesity-associated protein (FTO) serve as potential prognostic biomarkers for AIS. O’Connell et al. (2017) through sequencing analysis, indicated that peripheral blood A-kinase anchor protein 7 (AKAP7) mRNA may function as a biomarker for AIS, aiding in the identification of patients at high risk of blood-brain barrier (BBB) disruption following stroke. Additionally, previous studies have suggested that plasma microRNA-99b (miR-99b) levels represent a potential diagnostic and prognostic biomarker for AIS (Wu et al. 2020). Lu et al. (2023) confirmed that serum exosomal sirtuin 2 (SIRT2) is a potential diagnostic factor for AIS and a risk factor for higher National Institutes of Health Stroke Scale (NIHSS) scores. Collectively, these studies illustrate that the use of peripheral blood for detecting AIS biomarkers and exploring potential therapeutic targets is a feasible and promising approach.

Notable advancements have been achieved by research teams in the field of m^6^A and its associated proteins (e.g., METTL3 and METTL14). The stable complex formed by METTL3 and METTL14 in mammalian cells is pivotal for m^6^A modification process (Cui et al. 2024; Tang et al. 2024). A study conducted by Shen Hongjie et al. revealed that METTL14 can regulate the modification of bivalent chromatin domains in mouse embryonic stem cells, and this regulatory effect is independent of METTL3 and m^6^A modifications. However, the specific role of METTL14 in human AIS remains to be further elucidated (Mu et al. 2023).

This study shows that METTL14 is upregulated in AIS and has a negative correlation with GADD45B. Bioinformatics analysis and OGD-treated HUVEC transfection experiments indicate that METTL14 can directly act on GADD45B, and GADD45B has multiple high-confidence m^6^A modification sites. Since GADD45B is a recognized protective factor, it suggests that METTL14 is a risk factor for AIS. Therefore, reducing the level of METTL14 in AIS patients through regulation may have a good therapeutic effect, or directly upregulating GADD45B. The proposed biomarker in this study aims to supplement the existing CT/MRI imaging examinations. This biomarker detection has the advantages of minimal invasiveness (only requiring peripheral blood), low detection cost, and short time consumption. It is particularly suitable for primary medical institutions to conduct preliminary screening of suspected AIS populations, or to assist in the judgment of cases with unclear imaging examination results (Marquez et al. 2023). Meanwhile, this biomarker holds potential predictive value for the risk of AIS, providing a reference for early intervention in high-risk populations.

He et al. (2016) validated GADD45B is a pro-survival gene that silences its expression; specifically, they identified it as a critical mediator in ischemia/hypoxia-induced autophagic death of cortical neurons. GADD45B protects cortical neurons against cerebral ischemia-induced injury through multiple mechanisms, including regulation of autophagy and apoptosis. Tan et al (2021) explored the role of GADD45B in a MCAO model. Their findings showed that GADD45B-RNA interference (RNAi) reduced the proliferation and differentiation of neural cells in the subventricular zone (SVZ) at the infarct boundary following ischemic injury, which was accompanied by downregulated expression of BDNF. In contrast, environmental enrichment (EE) therapy after ischemic injury upregulates the expression of both GADD45B and BDNF and enhances neurogenesis in the SVZ. Notably, inhibition of GADD45B significantly impaired EE-induced neurogenic proliferation. These results confirm that GADD45B is closely associated with SVZ neurogenesis after AIS and that it mediates EE-induced neurogenesis via BDNF. In the present study, we experimentally demonstrated that the level of m^6^A-modified GADD45B mRNA was increased, whereas BDNF expression was downregulated in AIS . Combined with the aforementioned research findings, these results allow us to infer a potential regulatory axis: METTL14 may promote m^6^A modification of GADD45B mRNA; this elevated m^6^A modification is likely to reduce the stability of GADD45B mRNA, leading to its downregulation, which in turn results in the decreased expression of BDNF. In summary, GADD45B exerts multifaceted roles in AIS, as it not only acts as a pro-survival gene to protect neurons from injury, but also modulates neurogenesis and apoptosis by regulating factors such as BDNF. This highlights the complexity of GADD45B’s function in the pathophysiology of AIS and provides a potential molecular target for the development of therapeutic strategies against this disease. However, further in vivo validation and mechanistic exploration are required to fully clarify the regulatory details of the METTL14/GADD45B/BDNF axis and to support its translational application in clinical practice.

The team led by Esposito E found that, compared with sham operation and MCAO alone, MCAO plus postconditioning resulted in increased BDNF expression. This indicates that postconditioning after MCAO enhances the capacity of astrocytes to produce BDNF, BDNF is involved in neuroprotection, neurogenesis, and angiogenesis (Esposito et al. 2023). Zhu et al. (2023) demonstrated that BDNF-modified neural stem cell-derived exosomes (BDNF-hNSC-Exo) exhibited significant therapeutic effects in ischemic stroke models. The underlying mechanisms include the inhibition of microglial activation and promotion of the differentiation of endogenous neural stem cells into neurons. These findings suggest that BDNF plays a crucial role in facilitating neural repair and regeneration, and that the delivery of BDNF via neural stem cell-derived exosomes may represent an effective therapeutic strategy.

Bioinformatics analysis and OGD HUVECs transfection experiments in this study indicate that GADD45B may directly interact with BDNF. Correlation analysis revealed a positive correlation between GADD45B expression levels and BDNF in AIS. These findings suggest that BDNF is highly likely a key factor in AIS repair. Our analysis demonstrated a high diagnostic value of the METTL14/GADD45B/BDNF axis. After screening, METTL14, GADD45B, BDNF, GLOB, HDL-C, hypertension, diabetes, and BDNF protein levels were incorporated into the nomogram model, which showed good predictive performance. However, this study has several limitations. First, the sample size of 177 pairs was relatively small; therefore, multicenter, large-scale clinical studies are needed to enhance the credibility of the results. Second, certain AIS-related factors such as exercise habits, BMI, and sleep status were not collected. Addressing these limitations and deepening mechanistic insights will provide new support for precise prevention and control of AIS.

## Conclusion

Previous studies have shown AIS is a multifactorial disease, and the METTL14/GADD45B m^6^A methylation/BDNF axis represents a potentially key regulatory pathway. Specifically, METTL14 might indirectly regulate BDNF expression by modulating the methylation status of GADD45B. Collectively, these three genes show promise as biomarkers and therapeutic targets for AIS, and integrating them with conventional risk factors into a predictive model enables the effective assessment of AIS risk in both patients and healthy individuals, offering significant clinical benefits through timely intervention.

## Supplementary Information

Below is the link to the electronic supplementary material.

Supplementary Material 1

Supplementary Material 2

Supplementary Material 3

## Data Availability

No datasets were generated or analysed during the current study.
