# Supplementary material for: The Role and Diagnostic Efficacy of the METTL14/GADD45B m6A Methylation/BDNF Regulatory Axis in Acute Ischemic Stroke
Source: Cell Mol Neurobiol. 2026 Apr 16;46:119. doi: 10.1007/s10571-026-01710-0 (PMC13385536; doi:10.1007/s10571-026-01710-0)
Supplement: Supplementary file 1 — Supplementary Material 1 [file 10571_2026_1710_MOESM1_ESM.docx]

a


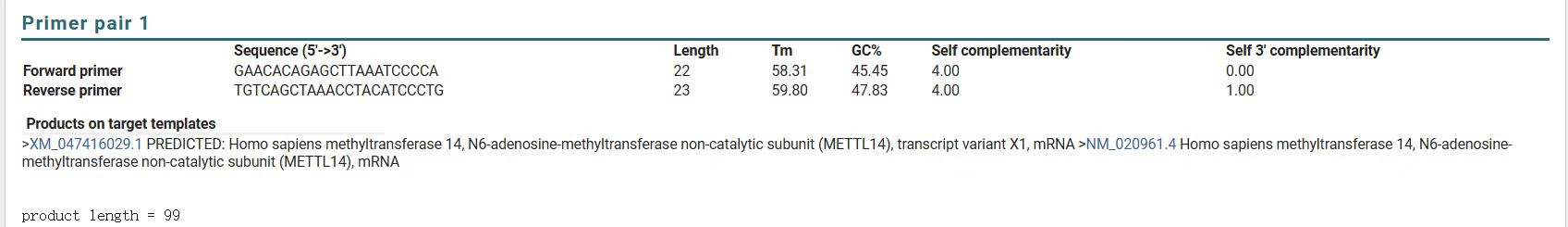


b


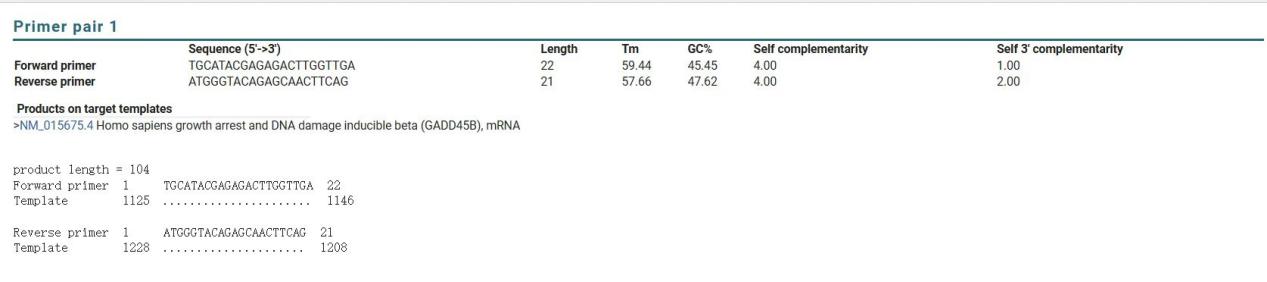


c


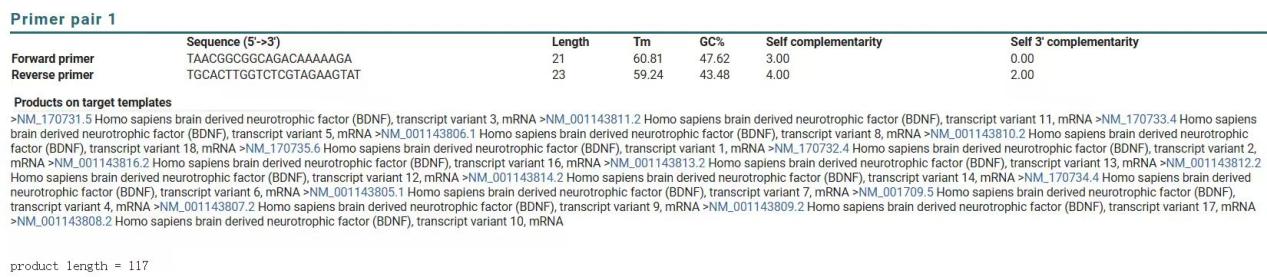


d


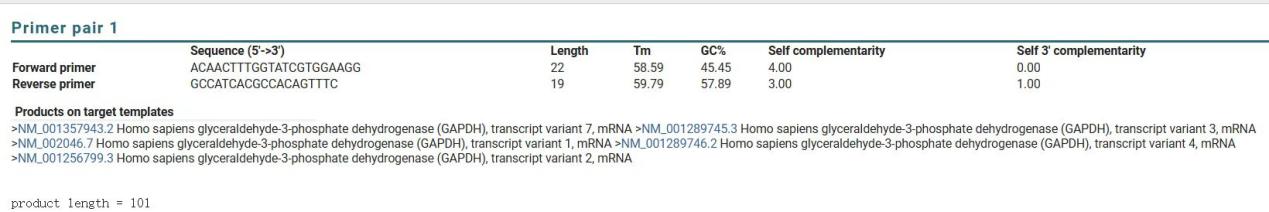


Primer specificity verification. a METTL14 primer specificity verification, b GADD45B primer specificity verification, c BDNF primer specificity verification, d GAPDH primer specificity verification.
