# Supplementary material for: The Role and Diagnostic Efficacy of the METTL14/GADD45B m6A Methylation/BDNF Regulatory Axis in Acute Ischemic Stroke
Source: Cell Mol Neurobiol. 2026 Apr 16;46:119. doi: 10.1007/s10571-026-01710-0 (PMC13385536; doi:10.1007/s10571-026-01710-0)
Supplement: Supplementary file 3 — Supplementary Material 3 [file 10571_2026_1710_MOESM3_ESM.pdf]

Validation Data

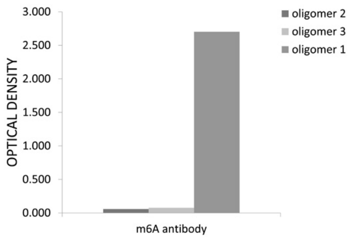

The m6A rabbit polyclonal antibody (4µg, A17924) are tested in Nucleotide Array against N6-methyladenosine (m6A) and unmodified adenosine (100pmol for each oligomer).
Oligomer 1 - N6-methyladenosine (m6A-CUGGUAACGAAUGGCU-G-Biotin)
Oligomer 2 - unmodified adenosine (ACUGGUAACGAAUGGCU-G-Biotin)
Oligomer 3 - unmodified adenosine (AAAAAAAAAAAAAAAAA-Biotin)

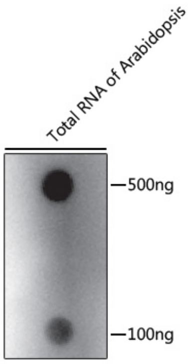

The m6A rabbit polyclonal antibody (500ng/100ng, A17924) are tested in Dot Blot against total RNA of Arabidopsis.
(This image is courtesy of an anonymous Abreview)

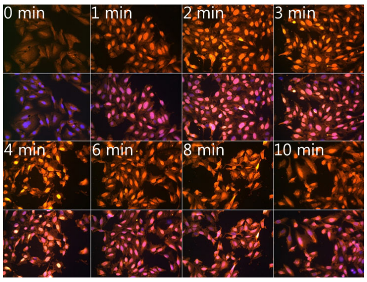

U2OS cells pre-treated with BrdU were subjected UVC irradiation incubated at 37 °C for the indicated time, and stained for m6A Polyclonal Antibody (A17924). DAPI, 4′,6-diamidino-2-phenylindole. Global UVC irradiation exceed cytoplasmic leavel,peaking at 2 min after irradiation and diminishing over the following 8 min.

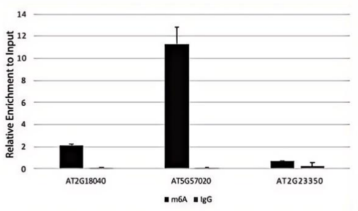

Immunoprecipitation analysis of 25µg total RNA extracts of Arabidopsis (25µg total RNA extracts of Arabidopsis was diluted to 200 µl IP buffer system) using 2ul m6A antibody (A17924).

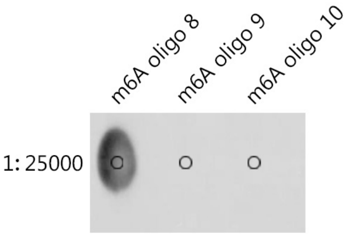

The m6A rabbit polyclonal antibody (A17924) are tested in Dot Blot against N6-methyladenosine (m6A) and unmodified adenosine.
Oligomer 8 - ATAACTGG-m6A-CCGAATGG
Oligomer 9 - ATAACTGGACCGAATGG
Oligomer 10 - AAAAAAAAAAAAAAAAA-biotin.
